# Development and Validation of a Reference Data Set for Assigning *Staphylococcus* Species Based on Next-Generation Sequencing of the 16S-23S rRNA Region

**DOI:** 10.3389/fcimb.2019.00278

**Published:** 2019-08-07

**Authors:** Maja Kosecka-Strojek, Artur J. Sabat, Viktoria Akkerboom, Karsten Becker, Evert van Zanten, Guido Wisselink, Jacek Miedzobrodzki, Anna M. D. (Mirjam) Kooistra-Smid, Alexander W. Friedrich

**Affiliations:** ^1^Department of Microbiology, Faculty of Biochemistry, Biophysics and Biotechnology, Jagiellonian University, Kraków, Poland; ^2^Department of Medical Microbiology, University of Groningen, University Medical Center Groningen, Groningen, Netherlands; ^3^Institute of Medical Microbiology, University Hospital Münster, Münster, Germany; ^4^Certe, Department of Medical Microbiology, Groningen, Netherlands

**Keywords:** NGS, *Staphylococcus*, 16S-23S rDNA, diagnostics, real-time PCR

## Abstract

Many members of the *Staphylococcus* genus are clinically relevant opportunistic pathogens that warrant accurate and rapid identification for targeted therapy. The aim of this study was to develop a careful assignment scheme for staphylococcal species based on next-generation sequencing (NGS) of the 16S-23S rRNA region. All reference staphylococcal strains were identified at the species level using Sanger sequencing of the 16S rRNA*, sodA, tuf*, and *rpoB* genes and NGS of the 16S-23S rRNA region. To broaden the database, an additional 100 staphylococcal strains, including 29 species, were identified by routine diagnostic methods, 16S rRNA Sanger sequencing and NGS of the 16S-23S rRNA region. The results enabled development of reference sequences encompassing the 16S-23S rRNA region for 50 species (including one newly proposed species) and 6 subspecies of the *Staphylococcus* genus. This study showed *sodA* and *rpoB* targets were the most discriminative but NGS of the 16S-23S rRNA region was more discriminative than *tuf* gene sequencing and much more discriminative than 16S rRNA gene sequencing. Almost all *Staphylococcus* species could be distinguished when the max score was 99.0% or higher and the sequence similarity between the best and second best species was equal to or >0.2% (min. 9 nucleotides). This study allowed development of reference sequences for 21 staphylococcal species and enrichment for 29 species for which sequences were publicly available. We confirmed the usefulness of NGS of the 16S-23S rRNA region by identifying the whole species content in 45 clinical samples and comparing the results to those obtained using routine diagnostic methods. Based on the developed reference database, all staphylococcal species can be reliably detected based on the 16S-23S rRNA sequences in samples composed of both single species and more complex polymicrobial communities. This study will be useful for introduction of a novel diagnostic tool, which undoubtedly is an improvement for reliable species identification in polymicrobial samples. The introduction of this new method is hindered by a lack of reference sequences for the 16S-23S rRNA region for many bacterial species. The results will allow identification of all *Staphylococcus* species, which are clinically relevant pathogens.

## Introduction

As of 2019, genus *Staphylococcus* comprises more than 50 validly described and proposed species, including both coagulase-positive staphylococci (CoPS) and coagulase-negative staphylococci (CoNS) (Euzéby, 1997; Becker et al., 2014; Parte, 2018). Moreover, an additional CoNS species (“*S. pseudolugdunensis*”) has been suggested (Tang et al., 2008).

The staphylococci are opportunistic pathogens that are a part of the natural microbiota of human and animal skin and mucous membranes (Kaspar et al., 2016; Islam et al., 2017; Mrochen et al., 2017; Kosecka-Strojek et al., 2018). However, changes in patient populations, such as the increased number of premature neonates, elderly and immunocompromised patients, and the increasing use of implanted foreign prosthetic material and indwelling catheters have led to a rise in documented infections caused by CoNS and CoPS other than *S. aureus* (Flores-Mireles et al., 2015; Giormezis et al., 2015; Butin et al., 2016; Savini et al., 2016). Because most studies report such infections as being caused by CoNS and do not differentiate isolates at the species level, the real impact of single species, especially less frequent species, is underreported. Moreover, species that have only been discovered in the last few years are not part of the routine diagnostic tests to identify bacterial species. Accurate identification is highly desirable for precise therapy, monitoring the spread of infections with epidemiologic characteristics and investigating disease progression (Ghebremedhin et al., 2008; Hwang et al., 2011; Shin et al., 2011; Becker et al., 2014).

In routine diagnostics, culture-dependent phenotypic tests, including automated systems such as the Vitek 2 (bioMérieux, La Balme Les Grottes, France) and BD Phoenix (BD Diagnostic Systems, Sparks, MD, USA), and matrix-assisted laser desorption ionization–time of flight mass spectrometry (MALDI-TOF MS), are used for identification of bacterial species. However, these methods are not always sufficiently reliable because of variable expression of phenotypic characteristics, and the databases are limited to only some species (Heikens et al., 2005; Dupont et al., 2010; Bergeron et al., 2011; Becker et al., 2015; Singhal et al., 2015; Ayeni et al., 2017; Gherardi et al., 2018). Accurate identification at the species level may not only change the diagnosis but can also help to identify unusual antimicrobial resistance patterns. The ideal method should have high discriminatory power and allow the identification of closely related species while also being relatively simple, inexpensive, rapid and reproducible. Therefore, genetic methods based on PCR or sequencing are good candidates for identification purposes. Most of these methods are based on specific nucleic acid target amplification and sequencing (Couto et al., 2001; Drancourt and Raoult, 2002; Becker et al., 2004).

For polymicrobial samples that need to be analyzed using culture-independent tests, the simultaneous identification of species of different genera using a single primer pair is a useful approach. As the 16S rRNA gene is universally present across bacteria, is highly conserved, and can be easily amplified using universal primers, microbial analyses are often performed using 16S rRNA amplicon sequencing (Nguyen et al., 2016). Although this method is widely used and accurate, the high degree of similarity between closely related species has limited its usefulness for identifying several CoNS species as well as distinguishing between the recently established species of the *S. aureus* complex (Hwang et al., 2011; Shin et al., 2011; Sabat et al., 2013; Tong et al., 2015).

Next-generation sequencing has highly improved microbiological genetic investigations by providing a cost-effective method to characterize bacterial genomes. The main advantage of NGS over Sanger sequencing is its ability to produce millions of reads in a single run. The NGS technologies produce reads with high sequence quality and high throughput, but the reads are short and need to be assembled *de novo*, which can be challenging; therefore, qualified investigators are highly desirable for NGS results analysis (Sabat et al., 2013). Recently, a NGS-based method for the 16S-23S rRNA region was developed by Sabat and colleagues (Sabat et al., 2017). This method is based on PCR amplification of the 16S-23S rRNA region, followed by amplicon sequencing on the MiSeq platform (Illumina, Inc., San Diego, CA, USA). The resulting reads are *de novo* assembled into contigs. Species identification is based on alignment of the contig sequences with sequences deposited in the reference databases (Sabat et al., 2017). This method can be used for identification of common pathogens, such as *Staphylococcus aureus* or *Escherichia coli*, directly from patient specimens with a high identification potential. Identification is also possible for non-cultured microorganisms, polymicrobial samples or samples with a DNA concentration that is too low for direct whole genome sequencing (WGS). However, the main disadvantage of this method is a lack of 16S-23S rRNA reference sequences for many bacterial species, which hinders proper interpretation of the results (Sabat et al., 2017). The main aim of this study was to develop a dataset of reference sequences of the 16S-23S rRNA region for almost all staphylococcal species. For validation of the dataset, we also compared the identification potential of NGS of the 16S-23S rRNA region with Sanger sequencing of the 16S rRNA, *sodA, tuf* and *rpoB* genes based on whole database and determined the cut off values for genus- and species-level identification of staphylococcal strains. Finally, we confirmed the applicability of NGS of the 16S-23S rRNA region for species identification with 45 clinical samples using the newly developed cut off values for staphylococcal species.

## Materials and Methods

### Bacterial Isolates

The collection of bacterial reference strains used in this study is described in Table 1. This collection included strains from 50 staphylococcal species (including one proposed species) and 6 subspecies. Most of the strains are deposited in reference microorganism collections, such as the Polish Collection of Microorganisms (PCM), Leibniz Institute DSMZ-German Collection of Microorganisms and Cell Cultures (DSMZ), American Type Culture Collection (ATCC), and Belgian Coordinated Collection of Microorganisms (BCCM). The collection with additional *Staphylococcus* strains is detailed in Supplementary Table 1. This collection included 101 strains originating from various human and animal infections and environmental strains. All strains were cultivated on blood agar medium with 5% sheep blood (bioMérieux, Marcy-l'Étoile, France) in 37°C for 20 h.

**Table 1:** *Staphylococcus* reference species and subspecies used for analyses.

**Species**	**Strain number in reference collection of microorganisms**	**Isolated from**	**Country/References**
*S. agnetis*	DSM 23656	Bovine mastitic milk	Finland
*S. argensis*	DSM 29875	River sediment	Germany
*S. argenteus*	DSM 28299	Blood culture of a 55-year-old Indigenous Australian female	Australia
*S. arlettae*	LMG 19113	Poultry skin	Belgium
*S. aureus*	PL408^a^	Natural environment	Poland
*S. auricularis*	PCM 2428	Human external ear	Country of origin unknown
*S. capitis subsp. capitis*	DSM 20326	Human skin	Country of origin unknown
*S. capitis subsp. urealyticus*	DSM 6717	Human skin	Country of origin unknown
*S. caprae*	DSM 20608	Goat milk	Country of origin unknown
*S. carnosus subsp. carnosus*	DSM 20501	Dry sausage	Country of origin unknown
*S. carnosus subsp. utilis*	DSM 11676	Fermented fish sauce (Pla-chom)	Thailand
*S. chromogenes*	PCM 2193	Skin of a healthy pig	Devriese et al., 1978; Hájek et al., 1986
*S. cohnii subsp. cohnii*	PCM 2108	Human skin	Schleifer and Kloos, 1975
*S. condimenti*	DSM 11674	Soy sauce mash	Japan
*S. delphini*	PCM 2407	Aqarium dolphin purulent skin lesion	Italy
*S. devriesei*	CCUG 58238T	Teat apex, healthy dairy heifer	Belgium
*S. epidermidis*	PCM 2532	Catheter	Winslow and Winslow, 1908; Evans, 1916
*S. equorum*	PCM 2487	Horse skin	Schleifer et al., 1984
*S. felis*	DSM 7377	Mass on the auricle in cat	Country of origin unknown
*S. fleurettii*	DSM 13212	Goat milk cheese	France
*S. gallinarum*	DSM 20610	Skin of chicken	Country of origin unknown
*S. haemolyticus*	PCM 2113	Human skin	Schleifer and Kloos, 1975
*S. hominis subsp. hominis*	DSM 20328	Human skin	Country of origin unknown
*S. hominis subsp. novobiosepticus*	ATCC 700236	Human blood	United States of America
*S. hyicus*	PCM 2192	Skin of pig with exudative epidermitis	Sompolinsky, 1953; Devriese et al., 1978
*S. intermedius*	DSM 20373	Pigeon nares	Country of origin unknown
*S. kloosii*	PCM 2440	Squirrel skin	Schleifer et al., 1984
*S. lentus*	PCM 2441	Goat udder	France
*S. lugdunensis*	PCM 2430	Axillary lymph node	France
*S. lutrae*	DSM 10244	Mammary gland of otter (*Lutra lutra*)	Scotland
*S. massiliensis*	CCUG 55927T	Human brain abscess	France
*S. microti*	DSM 22147	Liver of free-living common vole *Microtus arvalis*	Czech Republic
*S. muscae*	PCM 2406	Fly Stomomyx calcitrans	Czech Republic
*S. nepalensis*	DSM 15150	Nasal mucosa of a goat	Nepal
*S. pasteuri*	PCM 2445	Human vomit	France
*S. petrasii subsp. jettensis*	DSM 26618	Human blood	Belgium
*S. petrasii subsp. pragensis*	DSM 102853	Ejaculate (58-year-old patient with chronical prostatitis)	Czech Republic
*S. pettenkoferi*	DSM 19554	Human blood culture	Germany
*S. piscifermentans*	PCM 2409	Fermented shrimp	Thailand
*S. pseudintermedius*	LMG 22219T	Cat, lung tissue	Belgium
“*S. pseudolugdunensis”*	B006	Blood culture	United States of America
*S. rostri*	DSM 21968	Nasal cavity of a healthy pig	Switzerland
*S. saccharolyticus*	DSM20359	Plasma	Country of origin unknown
*S. saprophyticus subsp. bovis*	DSM 18669	Bovine nostril	Czech Republic
*S. saprophyticus subsp. saprophyticus*	DSM 100654	Cleanroom facility, TAS	Italy
*S. schleiferi subsp. schleiferi*	PCM 2426	Jugular catheter	Country of origin unknown
*S. schweitzeri*	DSM 28300	Nasal swab from a red-tailed monkey (*Cercopithecus ascanius*) within 12 h after the death of the animal	Gabon
*S. sciuri*	PCM 2424	Skin of eastern gray squirrel (*Sciurus carolinensis*)	Country of origin unknown
*S. simiae*	DSM 17636	Feces, South American squirrel monkey	Czech Republic
*S. simulans*	PCM 2106	Human skin	Kloos and Schleifer, 1975
*S. stepanovicii*	CCM 7717T	Animal vole	Poland
*S. succinus subsp. casei*	DSM 15096	Surface ripened cheese	Switzerland
*S. succinus subsp. succinus*	DSM 14617	Plant and soil inclusions within 25-35 million-year-old Dominican amber	Dominican Republic
*S. vitulinus*	PCM 2470	Ground lamb	Country of origin unknown
*S. warneri*	DSM 20316	Human skin	United States of America
*S. xylosus*	PL412^b^	Natural environment	Poland

a, b *Environmental isolates*.

### Clinical Samples

Forty-five clinical samples, collected in week 7 and 8 of 2018, from which the department of Medical Microbiology at Certe (Groningen, The Netherlands) cultured at least one *Staphylococcus* spp. were used. Bacterial identification was performed using the MALDI-TOF Vitek^®^ MS. The clinical samples consisted of; bronchoalveolar lavage (*n* = 1), catheter (*n* = 2), cervix/vagina post-partum (*n* = 2), insertion opening (*n* = 1), nose (*n* = 4), pus abscess (*n* = 2), pus wound (*n* = 3), sputum (*n* = 4), swab eye (*n* = 1), swab ulcer dig (*n* = 4), swab wound (*n* = 1); throat (*n* = 2), urine (*n* = 13), wound superficial (*n* = 4), and ear (*n* = 1).

### Genomic DNA Extraction

For genomic DNA extraction, the isolates were grown for 18–20 h at 37°C on blood agar plates. A full inoculation loop of 10 μl of bacterial colonies was homogenized with a TissueLyser II (Qiagen, Germantown, MD, USA). Total DNA was extracted by enzymatic lysis using the buffers and solutions provided with the DNeasy Blood and Tissue Kit (Qiagen, Germantown, MD, USA) according to the manufacturer's instructions. To obtain accurate quantification of the extracted genomic DNA for NGS, the Qubit dsDNA BR Assay Kit, which is a fluorometric method specific for duplex DNA, and the Qubit fluorometer 2.0 (Life Technologies, Inc., Eggenstein, Germany) were used according to the manufacturer's instructions.

### Genomic DNA Extraction of Clinical Samples

The Purelink Genomic DNA purification kit (Invitrogen, Carlsbad, CA, USA) was used for DNA extraction. Briefly, initial lysis was performed using 180 μl Purelink genomic digestion buffer and 20 μl Proteinase K. Digestion was performed in a thermoshaker at 56°C until lysis was complete. Two hundred microliter Purelink Genomic lysis/binding buffer was added to 200 μl of lysed sample and vortexed to create a homogenous solution. Two hundred microliter 96% ethanol was added and the DNA purification protocol was followed according to the manufacturer's instructions.

### PCR Amplification and Sanger Sequencing of the 16S rRNA, *sodA, tuf*, and *rpoB* Genes

All reference strains were identified at the species level by polymerase chain reaction (PCR) and Sanger sequencing of the 16S rRNA, *sodA, tuf*, and *rpoB* genes. Additional strains were identified at the species level by routine diagnostic methods and 16S rRNA gene sequencing. The amplification and sequencing primers and PCR conditions are listed in Table 2. All PCR products were resolved by electrophoresis using the 2200 TapeStation System (Agilent Technologies, Santa Clara, CA, USA) and then purified using the DNA Clean & Concentrator™-5 purification kit (Zymo Research, Irvine, CA, USA). The pair-end Sanger sequencing with forward and reverse strand sequencing was performed in GATC/ Eurofins Genomics company (Ebersberg, Germany).

**Table 2:** The amplification and Sanger sequencing primers and PCR conditions used for *Staphylococcus* species identification.

**Target gene**	**Amplification primers**	**PCR program**	**Sequencing primers**	**Amount of sequenced PCR product**	**References**
16S rRNA (1284-bp)	LPW57 (5′-AGTTTGATCCTGGCTCAG-3′) LPW58 (5′-AGGCCCGGGAACGTATTCAC-3′)	1.94°C for 2 min Steps 2–4 25 x 2. 94°C for 30 sec 3. 58°C for 30 sec 4. 72°C for 60 sec 5. 72°C for 5 min	LPW57, LPW58	250 ng	Woo et al., 2001
*sodA* (430-bp)	d1 (5′-CCITAYICITAYGAYGCIYTIGARCC-3′) d2 (5′-ARRTARTAIGCRTGYTCCCAIACRTC-3′)	1.95°C for 3 min Steps 2–4 35 x 2. 95°C for 30 sec 3. 40°C/43°C/46°C for 60 sec 4. 72°C for 90 sec 5. 72°C for 10 min	d1, d2	100 ng	Poyart et al., 2001
*tuf* (884-bp)	Tseq271 (5′-AAYATGATIACIGGIGCIGCICARATGCA-3′) Tseq1138 (5′-CCIACIGTICKICCRCCYTCRCG-3′)	1.95°C for 5 min Steps 2–4 35 x 2. 95°C for 30 sec 3. 55°C for 60 sec 4. 72°C for 90 sec 5. 72°C for 7 min	TSeq271, TSeq1138	200 ng	Martineau et al., 2001
*rpoB* (740-bp)	Staph rpoB 1418f (5′- CAATTCATGGACCAAGC−3′) Staph rpoB 3554r (5′-CCGTCCCAAGTCATGAAAC-3′)	1.94°C for 5 min Steps 2–4 35 x 2. 94°C for 30 sec 3. 52°C for 30 sec 4. 72°C for 60 sec 5. 72°C for 5 min	Staph rpoB 1418f, Staph rpoB 1975r (5′-GCIACITGITCCATACCTGT-3′) or Staph rpoB 1876r (5′-GAGTCATCITTYTCTAAGAATGG-3′)	250 ng	Mellmann et al., 2006

### *S. aureus* and 16S rDNA Real-Time PCR of Clinical Samples

In order to detect the presence of *Staphylococcus* DNA in clinical samples, two real-time PCR assays were performed as showed in Table 3. Firstly, the 16S rDNA assay was used to assess the bacterial load followed by the more specific *S. aureus* real-time PCR.

**Table 3:** The amplification primers, probes and real-time PCR conditions used for *Staphylococcus* DNA detection in clinical samples.

**Clinical samples real-time PCR target**	**Primers**	**Probes**	**PCR amplification mix**	**PCR program**	**References**
***S. aureus*** **(Sa442 fragment)**	SA442-forward (5′-CAATCTTTGTCGGTACACGATATTCT-3′) SA442-reverse (5′-CAACGTAATGAGATTTCAGTAGATAATACAAC-3′)	SA442-probe 1 (5′-FAM-CACGACTAAATAAACGCTCATTCGCGATTTT-BHQ1-3′)SA442-probe 2 (5′-FAM-CACGACTAAATAGACGCTCATTCGCAATTTT-BHQ1-3′)	1x TaqMan™ Advanced Master Mix (Thermo Fisher Scientific, Waltham, MA, USA)	Uracil-N-glycosylase incubation 50°C for 2 minPolymerase activation 95°C for 10 min40 x95°C for 15 sec60°C for 60 sec	Nijhuis et al., 2014
**16S rDNA**	16S rDNA_F27 (5′-AGAGTTTGATCMTGGCTCAG-3′) 16S rDNA_R1491 (5′-CGGYTACCTTGTTACGACTTC-3′)	16S rDNA_P535 (5′-FAM-CAGCCGCGGTAATA-MGBNFQ−3′)	1x TaqMan™ Fast Advanced Master Mix (Thermo Fisher Scientific, Waltham, MA, USA)	Uracil-N-glycosylase incubation 50°C for 2 minPolymerase activation 95°C for 2 min50 xDenaturation at 95°C for 10 secAnnealing at 55°C for 10 secExtension at 72°C for 90 sec	Schuurman et al., 2004

### Next-Generation Sequencing of the 16S-23S rRNA Region

Amplification of the 16S-23S rRNA region was performed using the primers 16S-27F (5′-AGAGTTTGATCMTGGCTCAG-3′) and 23S-2490R (5′-GACATCGAGGTGCCAAAC-3′) as described previously (Sabat et al., 2017). The PCR program for pure strains was as follows: initial denaturation for 2 min at 94°C, followed by 30 cycles of denaturation at 94°C for 30 sec, annealing at 66°C for 30 sec, and extension at 72°C for 120 sec and a final extension at 72°C for 5 min. For clinical samples, inclusion of a polymerase (MTP Taq DNA Polymerase, Sigma-Aldrich, St. Louis, MO, USA) recommended by the vendor for clinical use was essential because contamination occurred in the negative controls. To enhance the sensitivity of the PCR product, the number of PCR cycles was increased to 35. The PCR program used for clinical samples was as follows: initial denaturation for 2 min at 94°C, followed by 35 cycles of denaturation at 94°C for 30 sec, annealing at 66°C for 30 sec, and extension at 72°C for 120 sec and a final extension at 72°C for 5 min. The same program was used for the included controls. The obtained PCR products were purified and the DNA libraries were prepared with Nextera XT DNA Sample Preparation Kit (Illumina, Inc., San Diego, CA, USA) according to the manufacturer's instructions. The indexed libraries were pooled and loaded onto an Illumina MiSeq reagent cartridge using MiSeq reagent kit v3 and 600 cycles. The 2 × 300 bp sequencing was run on an Illumina MiSeq platform.

### Data Analysis

The Sanger sequencing results were analyzed using the Chromas software (Technelysium Pty Ltd., South Brisbane, Australia). The obtained sequences were analyzed using nucleotide BLAST (Basic Local Alignment Search Tool, http://www.ncbi.nlm.nih.gov/BLAST/) and aligned to the reference sequences deposited in the GenBank (v. 231.0; June 25, 2019) and leBIBI databases. The best and the second best species alignments were analyzed.According to the criteria developed by Sabat et al. (2017), the bacterial species were assigned when the similarity score was 99% or higher and the similarity score differences with the next closest species was ≥0.2%. Therefore, the identification at the species level using Sanger sequencing of the 16S rRNA (1284-bp), *sodA* (430-bp), *tuf* (884-bp), and *rpoB* (740-bp) gene fragments was considered as unambiguous for sequences different in at least 3, 2, 3, and 3 nucleotides, respectively. The identification at the species level using NGS of the whole 16S-23S rRNA region (4.3-kb) was considered as unambiguous for sequences different in at least 9 nucleotides. All sequences were compared with each other and to whole public database. The sequences were aligned in ClustalW (Larkin et al., 2007), and the phylogenetic trees were constructed using the neighbor-joining method (Saitou and Nei, 1987). The trees were drawn to scale, with branch lengths in the same units as those of the evolutionary distances used to infer the phylogenetic tree. The evolutionary distances were computed using the Jukes-Cantor method (Tamura et al., 2004) and were in the units of the number of base substitutions per site. All positions containing gaps were eliminated. The evolutionary analyses were conducted in MEGA7 (Kumar et al., 2016). The pairwise comparison of each pair of sequences was obtained using the CLC Genomics Workbench (Qiagen, Germantown, MD, USA) considering deletions as differences.

The NGS generated 17,000–210,000 sequencing reads for pure culture to obtain a minimum coverage of 1,000 per sample. The fastq files (Illumina MiSeq) with read lengths of 250 or 300 nucleotides were *de novo* assembled with the DNASTAR SeqMan NGen software (version 12.1.0; DNASTAR, Madison, WI, USA). During read assembly, reads shorter than 250 nucleotides were excluded. For most species, the minimum match percentage was 85% or 93% and the mer size was set as 31 nucleotides. The minimum matches of 94% were required for *S. agnetis, S. capitis* subsp*. capitis, S. pettenkoferi*, and *S. saprophyticus* subsp*. saprophyticus* and 86% for *S. carnosus* subsp*. utilis, S. haemolyticus, S. massiliensis*, and *S. nepalensis* to obtain the best quality sequences. After assembly, mean sample coverage was 4349.75-fold. However, the coverage per sample varied between 966.96- and 10649.1-fold. Only runs with a Q30 read quality score of >80% were accepted. If the assembly resulted in multiple contigs, the obtained ones were checked for length and quality in order to select the longest main contig with the highest reads amount assigned. Finally, the main contig was exported as fasta file for use in the subsequent analyses. For all species and subspecies, the main contig comprising the whole 16S-23S rRNA region extending from 4,237 (*S. epidermidis*) to 4,625 nucleotides (*S. lugdunensis*) was obtained. For clinical samples, the mer size was set as 31 nucleotides, and the minimum match percentage was 93%. Most of the identified bacterial species or genera were represented by a single contig of the expected size of approximately 4,500 bp, but in some cases smaller contigs ranging from 700 to 3,500 bp in size represented the same bacterial species in a sample. If several contigs were annotated to the same microorganism in some samples, the reads of these contigs were added up. Species identification was based on alignment of contig sequences with 16S-23S rRNA sequences deposited in the GenBank database using nucleotide BLAST and comparison to the leBIBI database (using the 16S rRNA gene sequence as a reference). Additionally, all staphylococcal species identifications were checked with the *Staphylococcus* reference database. The bacterial species and genus were assigned when the similarity scores were as previously described by Sabat et al. (2017). A score below 90% was interpreted as an unidentified organism.

### Nucleotide Sequence Accession Numbers

The 380 sequences for 56 *Staphylococcus* species and subspecies were annotated using the NCBI Sequin software and deposited in the GenBank database (http://www.ncbi.nlm.nih.gov/genbank/) under the following accession numbers: for the 16S-23S rRNA region, MF678861–MF678916 and MK015765-MK015864; for the 16S rRNA gene, MF678917-MF678972; for the *sodA* gene, MF679029-MF679084; for the *tuf* gene, MF678973-MF679028; and for the *rpoB* gene, MF679085-MF679140.

## Results

### Sanger Sequencing of the 16S rRNA Gene

As presented in Table 4, Sanger sequencing of the 16S rRNA gene allowed unambiguous identification at the species level for 27 species (54% of all species). Identification of the following pairs or groups of species was impossible because the 16S rRNA gene sequences were identical or almost identical (1 or 2 nucleotide differences): *S. aureus*- *S. argenteus*- *S. schweitzeri*; *S. argensis*- *S. pettenkoferi* – “*S. pseudolugdunensis”*; *S. pseudintermedius*- *S. intermedius*; *S. piscifermentans*- *S. carnosus* subsp. *carnosus*; *S. capitis* subsp. *urealyticus*- *S. caprae; S. agnetis – S. hyicus; S. condimenti - S. carnosus; S. epidermidis - S. haemolyticus; S. haemolyticus - S. equorum; S. gallinarum - S. haemolyticus; S. cohnii - S. kloosii; S. cohnii - S. nepalensis; S. pasteuri - S. warneri; S. microti - S. rostri; S. saprophyticus - S. xylosus; S. sciuri - S. lugdunensis* and *S. vitulinus - S. aureus*. The pairs of species *S. fleurettii*- *S. simulans, S. simulans*- *S. vitulinus* and *S. massiliensis*- *S. vitulinus* differed in the highest number of nucleotides, which equaled 57 for each pair of sequences. Most subspecies were not distinguishable by 16S rRNA gene sequencing. The only exception was the pair *S. petrasii* subsp. *jettensis* – *S. petrasii* subsp. *pragensis*, which differed by 6 nucleotides and could be easily identified.

**Table 4:** Summary of the performance of 16S rRNA, *sodA, rpoB, tuf* genes and 16S-23S rRNA region sequencing used for differentiation of *Staphylococcus* genus.

	**16S rRNA gene**	***sodA* gene**	***tuf* gene**	***rpoB* gene**	**NGS 16S-23S rRNA**
Unambiguous species identification	27 species (54%)	48 species (96%)	41 species (82%)	48 species (96%)	45 species (90%)
The lowest amount of nucleotides differences	0	0	0	0	0
The highest amount of nucleotides differences	57	149	120	192	667
No. of species without reference sequences in the databases	0	7	9	8	21

### Sanger Sequencing of the *rpoB* Gene

Sanger sequencing of the *rpoB* gene allowed unambiguous identification at the species level for 48 species (including one proposed species), which constituted 96% of the species. Identification of the following pairs or groups of species was impossible because the *rpoB* gene sequences were identical or almost identical: *S. felis - S. cohnii* and *S. intermedius - S. pseudintermedius*. The *rpoB* sequences for eight species (*S. argensis, S. chromogenes, S. devriesei, S. massiliensis, S. muscae*, “*S. pseudolugdunensis,” S. schweitzeri* and *S. stepanovicii*) were not available in the GenBank (v. 231.0; June 25, 2019) database. Identification at the subspecies level was possible for *S. hominis* subsp. *hominis*- *S. hominis* subsp. *novobiosepticus, S. petrasii* subsp*. jettensis*- *S. petrasii* subsp*. pragensis*, and *S. succinus* subsp*. casei*- *S. succinus* subsp*. succinus* using the *rpoB* gene. Considering the nucleotide differences in the *rpoB* gene sequences between pairs of species used within this study, the pairs with the lowest differences were *S. nepalensis*- *S. stepanovicii* (5 nucleotides) and *S. pettenkoferi* – “*S. pseudolugdunensis”* (13 nucleotides). The pair with the highest nucleotide difference was *S. fleurettii*- *S. piscifermentans*, which differed in 192 nucleotides and was the highest difference among those of all genes used in this study (Table 4).

### Sanger Sequencing of the *sodA* Gene

Sanger sequencing of the *sodA* gene allowed unambiguous identification at the species level for 48 species (including one proposed species), which constituted 96% of the species. Identification of the following pairs or groups of species was impossible because the *sodA* gene sequences were identical or almost identical: *S. nepalensis - S. hominis* and *S. warneri - S. epidermidis*. The *sodA* gene sequences were not available in the GenBank (v. 231.0; June 25, 2019) database for 7 species (*S. argensis, S. devriesei, S. petrasii*, “*S. pseudolugdunensis,” S. schweitzeri, S. simiae*, and *S. stepanovicii*) (Table 4). The pairs of species used within this study with the lowest nucleotide differences in the *sodA* gene sequences were *S. pettenkoferi* – “*S. pseudolugdunensis”* (4 nucleotides), *S. argensis* – “*S. pseudolugdunensis”* (6 nucleotides) and *S. argensis*- *S. pettenkoferi* (8 nucleotides), and the pair with the highest difference was *S. aureus*- *S. fleurettii*, which differed in 149 nucleotides (Table 4). Only the pair *S. petrasii* subsp*. jettensis* – *S. petrasii* subsp*. pragensis* could be identified at the subspecies level using *sodA* gene sequencing.

### Sanger Sequencing of the *tuf* Gene

The *tuf* gene sequencing allowed identification at the species level of 41 species, which accounted for 82% of all species. Due to sequence similarities with another species, identification was impossible for pairs: *S. aureus - S. schweitzeri; S. microti - S. rostri; S. pettenkoferi - “S. pseudolugdunensis”; S. pasteuri - S. warneri* and *S. vitulinus - S. sciuri*. For 9 species (*S. argensis, S. devriesei, S. microti, S. petrasii, S. piscifermentans, S. rostri, S. schweitzeri, S. simiae* and *S. stepanovicii*), the *tuf* gene reference sequences were not available in the GenBank (v. 231.0; June 25, 2019) database. The obtained results allowed the pairs with the lowest nucleotide differences [*S. delphini*- *S. pseudintermedius* (3 nucleotides), *S. carnosus*- *S. condimenti* (4 nucleotides), and *S. argenteus*- *S. aureus* (5 nucleotides)] and the pair with the highest difference [*S. auricularis*- *S. vitulinus* (120)] to be defined (Table 4). The *tuf* gene sequencing also allowed identification at the subspecies level for *S. hominis* subsp*. hominis*- *S. hominis* subsp*. novobiosepticus* and *S. petrasii* subsp*. jettensis*- *S. petrasii* subsp*. pragensis*.

### Next-Generation Sequencing of the 16S-23S rRNA Region

The nucleotide sequences of the 16S-23S rRNA region were obtained for all staphylococcal species. Analysis of the GenBank (v. 231.0; June 25, 2019) database showed that 16S-23S rRNA region sequences were available for 29 *Staphylococcus* species, whereas this study allowed the development of nucleotide sequences for an additional 21 species. Taking into consideration the differences in the length of the intergenic spacer located between the 16S and 23S rRNA genes, the average sequence length of the 16S-23S rRNA region was determined and equaled 4,381 nucleotides. The highest similarity among species analyzed within this study was found between *S. pettenkoferi* and “*S. pseudolugdunensis”* showing 99.8% sequence homology (7 nucleotides of difference), while the highest nucleotide difference was found between *S. delphini* and *S. fleurettii* and equaled 667 nucleotides (85.6% similarity).

### Comparison of the Sequencing Methods

All strains from the collection were characterized by Sanger sequencing of the 16S rRNA, *rpoB, sodA*, and *tuf* genes but identification to the species level was not possible by all targets used due to identical or almost identical sequence or the lack of some reference sequences in the GenBank (v. 231.0; June 25, 2019) database. The identification was confirmed by 2 targets for 4 species (*S. aureus, S. pettenkoferi*, “*S. pseudolugdunensis”* and *S. schweitzeri*), by 3 targets for 12 species and by 4 targets for the vast majority of the species (34 species) (Table 4).

**Figure 1:**
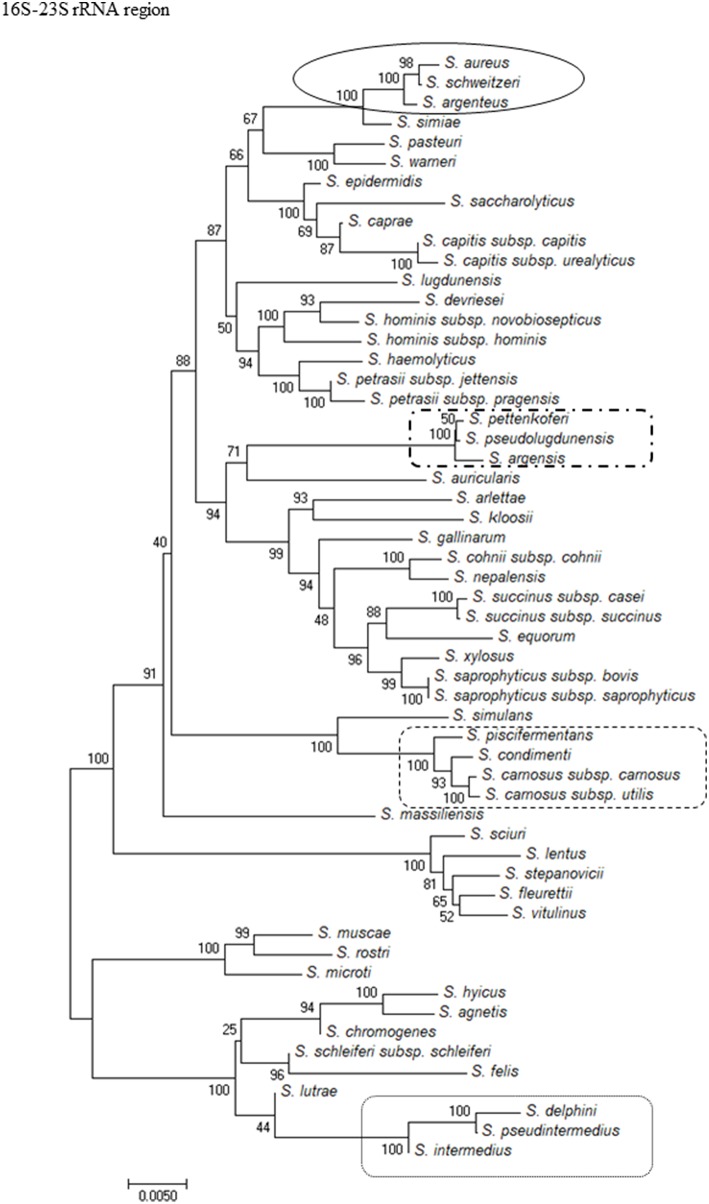
Evolutionary relationships of staphylococci species based on 16S-23S rRNA region. The evolutionary history was inferred using the Neighbor-Joining method. The percentage of replicate trees in which the associated taxa clustered together in the bootstrap test (1,000 replicates) are shown next to the branches. The tree is drawn to scale, with branch lengths in the same units as those of the evolutionary distances used to infer the phylogenetic tree. The evolutionary distances were computed using the Jukes-Cantor method and are in the units of the number of base substitutions per site. Evolutionary analyses were conducted in MEGA7. The strains which are placed in boxes have grouped together in all methods used.

To show the relationships among the species, phylogenetic trees were constructed. The computed overall mean distances according to the Jukes-Cantor model for the 16S rRNA, *sodA, tuf*, and *rpoB* genes and the 16S-23S rRNA region were 0.027, 0.023, 0.090, 0.178, and 0.045, respectively. Based on the criteria used for phylogenetic tree construction, the trees were constructed using the neighbor-joining method and revealed that the isolates clustered into groups for all of the methods used. All of the methods showed that *S. massiliensis* and *S. auricularis* were distantly related to the other species (Figure 1 and Supplementary Figures 1A–D). Analysis of the phylogenetic tree of the 16S-23S region showed similar clustering to the dendrogram based on 16S rRNA gene sequencing but was more discriminative, with unambiguous identification of almost all staphylococcal species (Figure 1).

For *Staphylococcus* species, *sodA* and *rpoB* genes Sanger sequencing had the highest identification potential allowing for an unambiguous identification of 96% of analyzed species, while the NGS-based method allowed for identification of 90% species (Table 4). The 16S rRNA gene Sanger sequencing had the lowest identification potential of all the methods used.

The nucleotides differences in the 16S-23S rRNA region sequences between pairs or species groups (*S. aureus*- *S. argenteus*- *S. schweitzeri, S. argensis*- *S. pettenkoferi* –“*S. pseudolugdunensis,” S. pseudintermedius*- *S. intermedius, S. piscifermentans*- *S. carnosus* subsp*. carnosus*, and *S. capitis* subsp*. urealyticus*- *S. caprae*) showed that all pairs or groups of species indistinguishable by Sanger sequencing of the 16S rRNA gene could be unambiguously identified with NGS of the 16S-23S rRNA region (Table 5).

**Table 5:** The comparison of 16S rRNA gene sequencing and NGS of 16S-23S rRNA region^a^.

**16S rRNA gene/16S-23S rRNA region**	***S. aureus***	***S. argenteus***	***S. argensis***	***S. pettenkoferi***	***S. capitis* subsp. *urealyticus***	***S. carnosus* subsp. *carnosus***	***S. intermedius***
*S. argenteus*	0/145						
*S. schweitzeri*	2/72	2/197					
*S. pettenkoferi*			1/80				
“*S. pseudolugdunensis”*			1/80	0/7			
*S. caprae*					2/253		
*S. piscifermentans*						2/32	
*S. pseudintermedius*							1/96

a *The nucleotides differences between the sequences of 16S rRNA gene and 16S-23S rRNA region for species used in this study are shown*.

### Additional Staphylococcal Strains

To compare the routine diagnostic methods with NGS of the 16S-23S rRNA region for identification of *Staphylococcus* species, 101 staphylococcal strains isolated from human infections, animal infections, natural environments and reference collections were included (Supplementary Table 1). All strains were identified at the species level by different standard diagnostic tools, such as MALDI-TOF MS, VITEK^®^ 2 ID, the BD Phoenix™ system and the API STAPH ID system. To confirm the obtained results, all strains were identified using 16S rRNA gene sequencing. Finally, all strains were identified by NGS of the 16S-23S rRNA region. Of all of the strains, 76 (76.8%) were correctly identified and 25 (25.2%) were misidentified by the routine diagnostic methods. The 16S rRNA sequencing allowed unambiguous identification of only 32 strains (31.7%), whereas 69 strains (68.3%) were incorrectly identified. NGS of the 16S-23S rRNA allowed unambiguous identification of all strains, including one non-staphylococcal strain misidentified by standard methods and also unidentified by 16S rRNA sequencing as *Shigella dysenteriae* or *Escherichia coli*. NGS of the 16S-23S region allowed unambiguous identification of 5 *S. pseudintermedius* strains, which was not possible with any other method used. Moreover, the reference 16S-23S rRNA sequence dataset allowed differentiation of one *S. agnetis* strain that was aligned as *S. hyicus* by MALDI-TOF MS and both 16S rRNA [1^st^ ID: *S. agnetis* 1283/1283 (100%); 2^nd^ ID: *S. hyicus* 1281/1283 (99.9%)] and 16S-23S rRNA region sequencing [1^st^ ID: *S. agnetis* 4422/4426 (99.9%); 2^nd^ ID: *S. hyicus* 4420/4426 (99.9%)] in comparison to GenBank (v. 231.0; June 25, 2019).

### Intraspecies Nucleotide Sequence Variation of the 16S-23S rRNA Region

To show the variability of 16S-23S rRNA region, the nucleotide sequence variation within *Staphylococcus* species was determined (Table 6). The analysis was performed for those species for which at least one nucleotide sequence of the 16S-23S rRNA region could be found in the GenBank (v. 231.0; June 25, 2019) database. For almost all species, the length of the 16S-23S rRNA region was the same within a species when the sequences obtained in this study and those deposited in GenBank (v. 231.0; June 25, 2019) were compared. The length of 16S-23S rRNA region was different within the same species only in case of *S. capitis* subsp*. capitis, S. muscae*, and *S. pettenkoferi*. The nucleotide variation within *Staphylococcus* species accounted from 0.05 to 5.9%, with the exception of *S. aureus, S. capitis*, and *S. sciuri* for which the intraspecies nucleotide variation was 9.70, 7.0, and 6.56%, respectively.

**Table 6:** The intraspecies polymorphism of 16S-23S rRNA region sequence within *Staphylococcus* genus.

**Species**	**The lowest intraspecies nucleotide difference compared to GenBank sequences**	**The highest intraspecies nucleotide difference compared to GenBank sequences**	**Amount of complete genome sequences/genome assembly/ 16S-23S rRNA region (GenBank)**	**16S-23S rRNA region length (bp)**	**Amount of 16S-23S rRNA region sequences deposited in GenBank after this study**
*S. agnetis*	2 (0.05%)	43 (0.97%)	1/0	4,427	2
*S. argensis*	–	–	0/0	4,364	1
*S. argenteus*	0 (0.00%)	14 (0.55%)	5/0	4,421	1
*S. arlettae*	–	–	0/0	4,316	3
*S. aureus*	0 (0.00%)	388 (9.70%)	365/78	4,389–4,589	4
*S. auricularis*	2 (0.05%)	2 (0.05%)	0/1	4,264	1
*S. capitis*	0 (0.00%)	322 (7.0%)	4/0	4,283–4,524	7
*S. caprae*	0 (0.00%)	169 (3.79%)	3/0	4,304–4,454	7
*S. carnosus*	0 (0.00%)	12 (0.28%)	2/0	4,288	3
*S. chromogenes*	–	–	0/0	4,410–4,323	2
*S. cohnii*	2 (0.05%)	101 (2.29%)	3/1	4,321–4,405	2
*S. condimenti*	0 (0.00%)	23 (1.44%)	2/1	4,288	1
*S. delphini*	0 (0.00%)	0 (0.00%)	0/1	4,578	1
*S. devriesei*	–	–	0/0	4,426	1
*S. epidermidis*	0 (0.00%)	257 (5.72%)	17/3	4,237–4,494	8
*S. equorum*	0 (0.00%)	7 (0.16%)	3/0	4,331	6
*S. felis*	1 (0.02%)	3 (0.07%)	1/0	4,431	2
*S. fleurettii*	–	–	0/0	4,300	2
*S. gallinarum*	–	–	0/0	4,322	3
*S. haemolyticus*	0 (0.00%)	271 (5.9%)	7/0	4,377–4,492	10
*S. hominis*	0 (0.00%)	180 (4.11%)	3/2	4,339–4,455	8
*S. hyicus*	0 (0.00%)	45 (1.02%)	1/2	4,422	1
*S. intermedius*	–	–	–	–	2
*S. kloosii*	1 (0.02%)	1 (0.02%)	1/0	4,442	1
*S. lentus*	–	–	0/0	4,328	3
*S. lugdunensis*	0 (0.00%)	144 (3.11%)	14/3	4,476–4,625	9
*S. lutrae*	4 (0.09%)	4 (0.09%)	1/0	4,267	1
*S. massiliensis*	–	–	0/0	4,277	1
*S. microti*	–	–	0/0	4,439	1
*S. muscae*	0 (0.00%)	30 (1.83%)	1/1	4,454	1
*S. nepalensis*	0 (0.00%)	92 (2.09%)	3/0	4,323–4,407	2
*S. pasteuri*	0 (0.00%)	150 (3.39%)	2/0	4,296–4,422	5
*S. petrasii*	–	–	0/0	4,328–4,457	2
*S. pettenkoferi*	19 (0.44%)	19 (0.44%)	1/0	4,299–4,317	2
*S. piscifermentans*	1 (0.02%)	1 (0.02%)	0/1	4,286	1
*S. pseudintermedius*	0 (0.00%)	180 (3.98%)	11/0	4,424–4,510	11
“*S. pseudolugdunensis”*	–	–	0/0	4,300	1
*S. rostri*	–	–	0/0	4,529	1
*S. saccharolyticus*	–	–	0/0	4,420	1
*S. saprophyticus*	0 (0.00%)	52 (3.22%)	5/2	4,326	5
*S. schleiferi*	0 (0.00%)	9 (0.21%)	6/0	4,293	1
*S. schweitzeri*	1 (0.02%)	1 (0.02%)	0/1	4,361	1
*S. sciuri*	0 (0.00%)	302 (6.56%)	2/1	4,295–4,505	6
*S. simiae*	3 (0.07%)	3 (0.07%)	0/1	4,331	1
*S. simulans*	0 (0.00%)	101 (2.31%)	6/2	4,287–4,380	4
*S. stepanovicii*	0 (0.00%)	0 (0.00%)	0/1	4,302	1
*S. succinus*	0 (0.00%)	6 (0.14%)	1/0	4,326	3
*S. vitulinus*	–	–	0/0	4,304	2
*S. warneri*	0 (0.00%)	101 (2.28%)	3/2	4,379–4,421	7
*S. xylosus*	0 (0.00%)	33 (0.76%)	4/1	4,331	4

### Clinical Samples

The aim of this part of our study was to assess the potential of NGS of the 16S-23S rRNA region to improve the resolution of *Staphylococcus* species identification directly from clinical samples. Forty-five clinical samples from various human infections were included (Table 7). Selection of these samples was based on a positive culture for *Staphylococcus* species. *Staphylococcus* species were identified by culture in all 45 samples, whereas NGS of the 16S-23S rRNA region identified *Staphylococcus* species in 37 samples (STA1-STA6, STA8-STA12, STA14-STA15, STA17-STA29, STA32-STA40, STA42, and STA45). Among the analyzed samples, conventional culture methods and NGS of the 16S-23S rRNA region identified the same *Staphylococcus* species in 27 samples (STA1, STA4, STA5, STA9- STA11, STA15, STA17- STA24, STA26- STA28, STA32- STA34, STA36- STA40, and STA42). In 10 samples, NGS of the 16S-23S rRNA region identified a higher number of *Staphylococcus* species than culture, showing two species in samples STA6, STA8, STA12, and STA45, three species in samples STA3, STA14, STA29, and STA35 and 4 and 6 species in samples STA2 and STA25, respectively. In a few samples, the culture-based methods allowed identification at the genus level, whereas NGS of the 16S-23S rRNA region was able to identify microorganisms at the species level (STA1, STA14, and STA31). Moreover, the NGS-based approach showed other pathogens and the coexisting microflora. The species content of the samples identified by NGS contained a range from one (STA4, STA26, and STA36) to a maximum of 33 (STA14) different microorganisms.

**Table 7:** Bacterial identification results from 45 clinical samples based on culture and NGS of 16S-23S rRNA region.

**Sample**	**Sample material**	**Species content by NGS of 16S-23S rRNA region (% of total reads)**	**Species content by conventional culture and MALDI-TOF Vitek MS identification (growth)**
STA1	Pus wound deep	*Streptococcus pyogenes* (83.6%), *Streptococcus dysgalactiae* (15.5%), *Streptococcus* sp. (0.6%), ***Staphylococcus aureus*** (0.2%)	***Staphylococcus aureus*** (large), *Streptococcus pyogenes* (large), *Streptococcus* group G (large)
STA2	Nose	***Staphylococcus aureus*** (65.3%), ***Staphylococcus epidermidis*** (15.2%), *Corynebacterium* sp. (6.4%), *Cutibacterium acnes* (4.4%), ***Staphylococcus lugdunensis*** (2.1%), *Finegoldia* sp. (1.6%), *Peptoniphilus* sp. (0.7%), *Streptococcus* sp. (0.5%), *Cutibacterium* sp. (0.5%), *Cutibacterium granulosum* (0.4%), ***Staphylococcus*** **sp**. (0.3%), *Micrococcus* sp. (0.3%), *Propionibacterium* sp. (0.3%), *Kocuria palustris* (0.2%), *Herbaspirillum* sp. (0.1%), Unidentified species (1.8%)	***Staphylococcus aureus*** (medium)
STA3	Nose	*Corynebacterium* sp. (32.9%), ***Staphylococcus aureus*** (7.8%), *Corynebacterium propinquum* (3.3%), *Streptococcus oralis* (1.9%), *Streptococcus canis* (1.6%), *Cutibacterium acnes* (1.5%), ***Staphylococcus epidermidis*** (0.8%), *Streptococcus* sp. (0.7%), *Neisseria sicca* (0.4%), *Actinomyces* sp. (0.4%), *Finegoldia* sp. (0.3%), *Veillonella* sp. (0.3%), *Corynebacterium pseudodiphtheriticum* (0.3%), *Herbaspirillum* sp. (0.2%), ***Staphylococcus*** **sp**. (0.1%), *Capnocytophaga* sp. (0.1%), *Enterococcus* sp. (0.1%), *Fusobacterium hwasookii* (0.1%), *Anaerococcus* sp. (0.1%), Unidentified species (47.0%), eukaryotic DNA (0.1%)	***Staphylococcus aureus*** (few)
STA4	Swab ulcus dig.	***Staphylococcus aureus*** (100%)	***Staphylococcus aureus*** (medium)
STA5	Swab ulcus dig.	***Staphylococcus aureus*** (59.6%), *Streptococcus dysgalactiae* (23.7%), *Streptococcus* sp. (3.1%), *Streptococcus pneumoniae* (2.7%), *Herbaspirillum* sp. (1.8%), *Sphingomonas sanguinis* (0.7%), *Gemella haemolysans* (0.3%), eukaryotic DNA (8.2%)	***Staphylococcus aureus*** (medium)
STA6	Pus abscess	***Staphylococcus aureus*** (95.5%), ***Staphylococcus capitis*** (0.8%), *Cutibacterium acnes* (0.6%), eukaryotic DNA (3.2%)	***Staphylococcus aureus*** (medium)
STA7	Wound supperficial	*Finegoldia* sp. (45.6%), *Anaerococcus tetradius* (7.6%), *Peptoniphilus harei* (7.4%), *Peptoniphilus coxii* (7.2%), *Streptococcus* sp. (6.8%), *Prevotella* sp. (3.2%), *Streptococcus agalactiae* (2.1%), *Anaerococcus obesiensis* (1.9%), *Prevotella disiens* (1.6%), *Anaerococcus* sp. (0.7%), Unidentified species (16.0%)	***Staphylococcus aureus*** (few), *Streptococcus agalactiae* (medium)
STA8	Pus wound deep	***Staphylococcus epidermidis*** (71.8%), ***Staphylococcus lugdunensis*** (17.7%), *Streptococcus* sp. (4.2%), *Streptococcus parasanguinis* (2.0%), *Herbaspirillum* sp. (1.0%), *Streptococcus mitis* (0.5%), *Sphingomonas sanguinis* (0.5%), *Streptococcus canis* (0.3%), eukaryotic DNA (2.0%)	***Staphylococcus lugdunensis*** (few)
STA9	Swab wound deep	*Streptococcus agalactiae* (28.3%), ***Staphylococcus aureus*** (24.3%), *Peptoniphilus harei* (12.7%), *Finegoldia magna* (7.3%), *Anaerococcus* sp. (6.7%), *Peptoniphilus coxii* (4.8%), *Anaerococcus vaginalis* (2.9%), *Fastidiosipila* sp. (2.8%), *Porphyromonas bennonis* (0.6%), *Actinotignum* sp. (0.6%), *Corynebacterium* sp. (0.4%), *Varibaculum cambriense* (0.2%), *Fastidiosipila sanguinis* (0.2%), *Cutibacterium acnes* (0.1%), ***Staphylococcus capitis*** (0.1%), *Streptococcus* sp. (0.1%), Unidentified species (7.5%), eukaryotic DNA (0.4%)	***Staphylococcus aureus*** (medium), *Streptococcus agalactiae* (few)
STA10	Wound supperficial	***Staphylococcus epidermidis*** (81.9%), *Herbaspirillum* sp. (5.6%), *Sphingomonas sanguinis* (5.4%), *Streptococcus thermophilus* (2.2%), *Paracoccus* sp. (1.1%), Unidentified species (0.7%), eukaryotic DNA (3.0%)	***Staphylococcus epidermidis*** (few)
STA11	Nose	***Staphylococcus epidermidis*** (45.2%), *Psychrobacter alimentarius* (29.3%), *Corynebacterium accolens* (15.2%), *Peptoniphilus* sp. (2.9%), *Anaerococcus* sp. (2.5%), *Corynebacterium* sp. (1.3%), *Cutibacterium granulosum* (1.1%), *Corynebacterium pseudodiphtheriticum* (0.7%), *Propionibacterium* sp. (0.3%), *Sphingomonas sanguinis* (0.1%), *Kocuria rhizophila* (0.1%), *Herbaspirillum* sp. (0.1%), Unidentified species (1.1%)	***Staphylococcus epidermidis*** (few)
STA12	Swab ulcus dig.	***Staphylococcus aureus*** (55.0%), *Streptococcus oralis* (28.2%), *Pseudomonas aeruginosa* (10.1%), *Finegoldia magna* (4.9%), *Morganella morganii* (0.7%), *Streptococcus pneumoniae* (0.3%), *Herbaspirillum* sp. (0.2%), *Anaerococcus* sp. (0.2%), ***Staphylococcus*** **sp**. (0.1%), Unidentified species (0.2%)	***Staphylococcus aureus*** (medium), *Pseudomonas aeruginosa* (medium)
STA13	Cervix/vagina post partum	*Megasphaera* sp. (33.8%), *Atopobium* sp. (6.4%), *Lactobacillus* sp. (3.8%), *Aerococcus* sp. (1.5%), *Peptostreptococcus* sp. (1.4%), ***Staphylococcus*** **sp**. (0.9%), *Anaerococcus tetradius* (0.3%), *Prevotella* sp. (0.3%), *Peptoniphilus* sp. (0.3%), *Gemella asaccharolytica* (0.2%), *Arcanobacterium* sp. (0.2%), *Streptococcus* sp. (0.2%), *Finegoldia* sp. (0.2%), *Dialister* sp. (0.2%), *Corynebacterium* sp. (0.1%), Unidentified species (50.3%)	***Staphylococcus haemolyticus*** (sporadic), ***Staphylococcus epidermidis*** (large)
STA14	Throat	*Faecalibacterium prausnitzii* (10.8%), *Streptococcus salivarius* (5.9%), ***Staphylococcus epidermidis*** (4.9%), *Oscillibacter* sp. (3.2%), *Bacteroides vulgatus* (2.9%), *Bacteroides uniformis* (2.6%), *Alistipes* sp. (2.0%), ***Staphylococcus lugdunensis*** (1.9%), *Lactobacillus* sp. (1.7%), *Escherichia coli* (1.7%), *Roseburia* sp. (1.6%), *Eubacterium* sp. (1.2%), *Barnesiella* sp. (1.0%), *Enterococcus faecium* (0.9%), *Eubacterium rectale* (0.8%), *Acutalibacter* sp. (0.7%), *Porphyromonas bennonis* (0.7%), *Monoglobus pectinilyticus* (0.5%), *Longibaculum* sp. (0.5%), *Blautia* sp. (0.5%), *Eubacterium siraeum* (0.5%), *Parabacteroides distasonis* (0.4%), ***Staphylococcus*** **sp**. (0.4%), *Bacteroides* sp. (0.4%), *Ruminococcus* sp. (0.3%), *Peptoniphilus* sp. (0.3%), *Herbaspirillum* sp. (0.3%), *Dialister* sp. (0.3%), *Intestinimonas* sp. (0.3%), *Parabacteroides* sp. (0.3%), *Sphingomonas zeae* (0.2%), *Colidextribacter massiliensis* (0.2%), *Odoribacter splanchnicus* (0.2%), Unidentified species (45.0%), eukaryotic DNA (4.6%)	*Escherichia coli* (medium), ***Staphylococcus*** **spp**. (few)
STA15	Urine	*Actinotignum* sp. (68.2%), ***Staphylococcus epidermidis*** (31.1%), *Actinotignum schaalii* (0.1%), Unidentified species (0.6%)	***Staphylococcus epidermidis*** (≥10e5 CFU/ml.)
STA16	Urine	*Lactobacillus* sp. (98.5%), *Lactobacillus iners* (0.8%), Unidentified species (0.7%)	***Staphylococcus haemolyticus*** (≥10e5 CFU/ml.)
STA17	Sputum	*Prevotella melaninogenica* (76.9%), ***Staphylococcus haemolyticus*** (10.0%), *Leptotrichia* sp. (5.7%), *Veillonella* sp. (0.1%), *Prevotella jejuni* (0.1%), *Prevotella* sp. (0.1%), Unidentified species (7.1%)	***Staphylococcus haemolyticus*** (medium)
STA18	Cathether	***Staphylococcus epidermidis*** (90.8%), *Enterococcus faecalis* (4.1%), *Enterococcus hirae* (1.7%), *Herbaspirillum* sp. (1.2%), *Sphingomonas sanguinis* (0.9%), *Roseateles* sp. (0.5%), *Ralstonia insidiosa* (0.2%), eukaryotic DNA (0.6%)	***Staphylococcus epidermidis*** (≥ 15 CFU), *Enterococcus faecium* (1 CFU)
STA19	Urine	***Staphylococcus epidermidis*** (91.9%), *Herbaspirillum* sp. (5.6%), *Sphingomonas sanguinis* (1.6%), *Streptococcus oralis* (0.6%), *Rhizobacter* sp. (0.2%), *Delftia acidovorans* (0.2%)	***Staphylococcus epidermidis*** (≥10e5 CFU/ml.)
STA20	Cathether	***Staphylococcus epidermidis*** (76.4%), *Herbaspirillum* sp. (8.4%), *Janthinobacterium svalbardensis* (2.0%), *Sphingomonas sanguinis* (1.2%), ***Staphylococcus capitis*** (1.2%), *Roseateles* sp. (0.7%), *Corynebacterium accolens* (0.5%), *Pseudomonas* sp. (0.4%), *Rhizobacter* sp. (0.3%), Unidentified species (2.3%), eukaryotic DNA (6.5%)	***Staphylococcus capitis*** (≥15 CFU),
			***Staphylococcus epidermidis*** (≥15 CFU)
STA21	Urine	***Staphylococcus epidermidis*** (72.5%), *Bacteroides dorei* (20.2%), *Bacteroides fragilis* (6.6%), Unidentified species (0.8%)	***Staphylococcus epidermidis*** (≥10e5 CFU/ml.)
STA22	Urine	***Staphylococcus haemolyticus*** (65.1%), *Dialister* sp. (4.2%), *Actinomyces* sp. (1.6%), *Solobacterium moorei* (1.5%), *Cutibacterium acnes* (1.1%), *Peptoniphilus lacrimalis* (1.1%), *Prevotella* sp. (0.9%), *Actinotignum* sp. (0.9%), *Porphyromonas bennonis* (0.7%), *Herbaspirillum* sp. (0.7%), *Eubacterium saphenum* (0.5%), *Peptoniphilus grossensis* (0.5%), *Peptoniphilus urinimassiliensis* (0.4%), *Kallipyga* sp. (0.4%), *Eubacterium* sp. (0.4%), *Jonquetella anthropic* (0.3%), *Peptoniphilus koenoeneniae* (0.3%), *Actinomyces turicensis* (0.3%), *Criibacterium bergeronii* (0.2%), *Ezakiella* sp. (0.2%), *Prevotella colorans* (0.2%), *Roseateles* sp. (0.2%), *Sphingomonas sanguinis* (0.2%), *Moryella indoligenes* (0.1%), *Howardella* sp. (0.1%), Unidentified species (17.8%)	***Staphylococcus heamolyticus*** (≥10e4 CFU/ml.)
STA23	Urine	***Staphylococcus saprophyticus*** (96.9%), *Veillonella* sp. (1.1%), *Streptococcus agalactiae* (0.8%), *Herbaspirillum* sp. (0.2%), *Moraxella osloensis* (0.2%), *Streptococcus* sp. (0.2%), *Streptococcus mitis* (0.2%), *Acidovorax* sp. (0.1%), *Brevundimonas vesicularis* (0.1%)	***Staphylococcus saprophyticus*** (≥10e4 CFU/ml.)
STA24	Swab eye	*Cutibacterium acnes* (47.5%), *Corynebacterium* sp. (9.7%), ***Staphylococcus epidermidis*** (4.4%), *Streptococcus sanguinis* (4.3%), *Herbaspirillum* sp. (4.2%), *Simonsiella* sp. (1.6%), *Veillonella* sp. (1.3%), *Sphingomonas sanguinis* (1.2%), *Corynebacterium jeikeium* (1.2%), *Roseateles* sp. (0.9%), *Gordonia* sp. (0.8%), *Fusobacterium nucleatum* (0.8%), *Finegoldia* sp. (0.6%), *Aquabacterium* sp. (0.5%), *Kocuria palustris* (0.5%), *Methylobacterium* sp. (0.4%), *Kocuria rhizophila* (1.5%), *Propionibacterium acnes* (0.4%), eukaryotic DNA (18.2%)	***Staphylococcus epidermidis*** (unknown)
STA25	Nose	***Staphylococcus epidermidis*** (69.3%), *Finegoldia magna* (7.9%) *Sphingomonas sanguinis* (3.6%), *Herbaspirillum* sp. (3.4%), *Pseudomonas fluorescens* (2.4%), ***Staphylococcus haemolyticus*** (1.8%), *Streptococcus sanguinis* (1.6%),	***Staphylococcus pasteuri*** (unknown)
		*Enterococcus faecalis* (1.5%), ***Staphylococcus*** **sp**. (1.2%), *Kocuria palustris* (1.0%), ***Staphylococcus hominis*** (0.7%), *Niastella* sp. (0.6%), ***Staphylococcus pasteuri*** (0.6%), *Corynebacterium* sp. (0.4%), *Propionibacterium acnes* (0.4%), ***Staphylococcus capitis*** (0.3%), *Janthinobacterium* sp. (0.2%), *Roseateles* sp. (0.2%), *Campylobacter concisus* (0.2%), *Streptococcus* sp. (0.2%), *Actinomyces neuii* (0.2%), Unidentified species (1.5%), eukaryotic DNA (0.8%)	
STA26	Urine	***Staphylococcus lugdunensis*** (100%)	***Staphylococcus lugdunensis*** (≥10e5 CFU/ml.)
STA27	Urine	***Staphylococcus epidermidis*** (97.3%), *Pseudomonas putida* (2.6%), Unidentified species (0.1%)	***Staphylococcus epidermidis*** (≥10e5 CFU/ml.)
STA28	Urine	*Aerococcus urinae* (95.7%), ***Staphylococcus*** **sp**. (3.6%), Unidentified species (0.7%)	***Staphylococcus epidermidis*** (≥10e5 CFU/ml.), *Aerococcus urinae* (≥10e5 CFU/ml.)
STA29	Pus wound deep	***Staphylococcus haemolyticus*** (43.1%), *Herminiimonas* sp. (11.2%), *Propionibacterium acnes* (9.2%), *Sphingomonas sanguinis* (6.6%), ***Staphylococcus epidermidis*** (6.3%), *Paracoccus yeei* (3.4%), *Janthinobacterium* sp. (1.7%), *Herbaspirillum* sp. (1.6%), *Pseudomonas* sp. (0.3%), *Pseudomonas lurida* (0.3%), ***Staphylococcus aureus*** (0.3%), Unidentified species (0.6%), eukaryotic DNA (15.5%)	***Staphylococcus haemolyticus*** (few), ***Staphylococcus epidermidis*** (few)
STA30	Insertion opening	*Corynebacterium* sp. (52.0%), *Peptoniphilus harei* (31.5%), *Corynebacterium tuberculostearicum* (1.6%), *Cutibacterium acnes* (1.1%), *Anaerococcus* sp. (0.7%), *Finegoldia* sp. (0.7%), *Herbaspirillum* sp. (0.5%), *Corynebacterium pseudogenitalium* (0.2%), *Herbaspirillum rubrisubalbicans* (0.1%), Unidentified species (11.5%), eukaryotic DNA (0.1%)	***Staphylococcus hominis*** (few), *Corynebacterium tuberculostearicum* (Medium)
STA31	Wound supperficial	*Streptococcus dysgalactiae* (98.8%), *Corynebacterium* sp. (0.5%), *Finegoldia* sp. (0.4%), Unidentified species (0.3%)	***Staphylococcus aureus*** (medium), *Streptococcus group C* (large)
STA32	Throat	***Staphylococcus aureus*** (98.4%), *Corynebacterium striatum* (1.0%), *Peptostreptococcus anaerobius* (0.4%), *Streptococcus* sp. (0.2%)	***Staphylococcus aureus*** (medium)
STA33	Ear	*Finegoldia* sp. (37.6%), *Gemella haemolysans* (11.5%), *Anaerococcus* sp. (7.2%), *Streptococcus pneumoniae* (4.0%), ***Staphylococcus*** **sp**. (2.3%), *Enterococcus* sp. (1.9%), *Peptoniphilus harei* (1.2%), *Prevotella* sp. (1.0%), *Eikenella corrodens* (0.9%), *Corynebacterium* sp. (0.6%), ***Staphylococcus aureus*** (0.4%), *Auricoccus* sp. (0.4%), *Prevotella melaninogenica* (0.3%), Unidentified species (29.9%), eukaryotic DNA (0.9%)	***Staphylococcus aureus*** (large)
STA34	Urine	***Staphylococcus aureus*** (68.7%), *Peptostreptococcus anaerobius* (7.6%), *Anaerococcus tetradius* (2.2%), *Anaerococcus* sp. (1.4%), *Peptoniphilus harei* (0.6%), *Corynebacterium simulans* (0.5%), *Herbaspirillum* sp. (0.2%), *Sphingomonas sanguinis* (0.2%), *Corynebacterium* sp. (0.2%), *Pedobacter* sp. (0.2%), Unidentified species (16.9%), eukaryotic DNA (1.8%)	***Staphylococcus aureus*** (≥ 10e5 CFU/ml.)
STA35	Urine	***Staphylococcus aureus*** (98.4%), ***Staphylococcus haemolyticus*** (1.0%), ***Staphylococcus epidermidis*** (0.5%)	***Staphylococcus aureus*** (≥ 10e5 CFU/ml.), *Candida parapsilosis* (10e4 CFU/ml.)
STA36	Urine	***Staphylococcus aureus*** (100%)	***Staphylococcus aureus*** (≥ 10e5 CFU/ml.)
STA37	Sputum	***Staphylococcus aureus*** (62.4%), *Streptococcus* sp. (16.6%), *Fusobacterium* sp. (5.2%), *Fusobacterium nucleatum* (3.6%), *Veillonella atypica* (3.2%), *Gemella morbillorum* (1.3%), *Prevotella* sp. (0.9%), *Prevotella conceptionensis* (0.6%), *Prevotella melaninogenica* (0.5%), *Parvimonas micra* (0.5%), *Streptococcus salivarius* (0.4%), *Capnocytophaga* sp. (0.2%), *Lactobacillus gasseri* (0.2%), *Streptococcus parasanguinis* (0.2%), Unidentified species (1.4%), eukaryotic DNA (2.8%)	***Staphylococcus aureus*** (large)
STA38	Urine	*Klebsiella* sp. (61.5%), *Pseudomonas* sp. (29.6%), ***Staphylococcus aureus*** (2.7%), *Stenotrophomonas* sp. (0.6%), *Acinetobacter* sp. (0.3%), *Enterobacter* sp. (0.3%), *Klebsiella oxytoca* (0.3%), *Pseudomonas azotoformans* (0.1%), Unidentified species (4.7%)	***Staphylococcus aureus*** (≥ 10e5 CFU/ml.), *Klebsiella oxytoca* (≥ 10e5 CFU/ml.)
STA39	Sputum	*Streptococcus anginosus* (32.0%), *Prevotella* sp. (26.9%), *Moraxella catarrhalis* (13.9%), ***Staphylococcus aureus*** (8.5%), *Prevotella oris* (3.5%), *Solobacterium moorei* (1.6%), *Streptococcus* sp. (1.1%), *Peptoniphilus harei* (0.6%), *Corynebacterium* sp. (0.5%), *Parvimonas micra* (0.4%), *Finegoldia* sp. (0.3%), *Streptococcus oralis* (0.3%), *Pseudomonas* sp. (0.1%), Unidentified species (9.1%), eukaryotic DNA (1.3%)	***Staphylococcus aureus*** (large), *Pseudomonas aeruginosa* (medium), *Klebsiella oxytoca* (few)
STA40	Sputum	***Staphylococcus aureus*** (8.5%), *Leptotrichia* sp. (2.3%), *Streptococcus thermophilus* (1.7%), *Herbaspirillum* sp. (1.5%), eukaryotic DNA (85.9%)	***Staphylococcus aureus*** (few), *Candida albicans* (few)
STA41	BAL	*Leptotrichia* sp. (37.4%), *Prevotella* sp. (13.5%), *Streptococcus* sp. (10.7%), *Veillonella atypica* (5.2%), *Fusobacterium* sp. (1.4%), *Selenomonas* sp. (1.3%), *Actinomyces* sp. (0.8%), *Parvimonas* sp. (0.8%), *Megasphaera* sp. (0.7%), *Atopobium* sp. (0.6%), *Streptococcus salivarius* (0.5%), *Tannerella* sp. (0.3%), *Lactobacillus* sp. (0.3%), *Cryptobacterium* sp. (0.3%), *Porphyromonas* sp. (0.3%), *Aerococcus* sp. (0.2%), *Prevotella melaninogenica* (0.2%), *Alloprevotella tannerae* (0.2%), *Dialister* sp. (0.2%), *Dialister pneumosintes* (0.2%), *Fretibacterium fastidiosum* (0.2%), *Mogibacterium* sp. (0.2%), *Stomatobaculum longum* (0.2%), *Centipeda* sp. (0.2%), *Peptostreptococcus* sp. (0.1%), *Prevotella nigrescens* (0.1%), *Streptococcus* mitis (0.1%), *Actinomyces odontolyticus* (0.1%), *Anaeroglobus* sp. (0.1%), *Atopobium parvulum* (0.1%), *Solobacterium* sp. (0.1%), *Eubacterium* sp. (0.1%), Unidentified species (23.6%)	***Staphylococcus aureus*** (few), *Aspergillus fumigatus* (few)
STA42	Pus abscess labia	***Staphylococcus aureus*** (30.8%), *Mobiluncus curtisii* (5.1%), *Dialister* sp. (1.8%), *Anaerococcus* sp. (1.7%), *Actinomyces* sp. (1.4%), *Peptoniphilus coxii* (1.1%), *Herbaspirillum* sp. (0.8%), *Peptoniphilus* sp. (0.6%), *Peptoniphilus harei* (0.6%), *Peptostreptococcus anaerobius* (0.5%), Unidentified species (16.7%), eukaryotic DNA (38.7%)	***Staphylococcus aureus*** (medium)
STA43	Cervix/vagina post partum	*Lactobacillus* sp. (50.7%), *Prevotella* sp. (17.4%), *Parvimonas* sp. (5.7%), *Streptococcus anginosus* (1.3%), *Gemella* sp. (1.1%), *Solobacterium* sp. (0.7%), *Anaerococcus obesiensis* (0.4%), *Peptoniphilus* sp. (0.3%), *Lactobacillus iners* (0.2%), *Solobacterium moorei* (0.1%), Unidentified species (22.1%), eukaryotic DNA (0.1%)	***Staphylococcus aureus*** (large), *Escherichia coli* (large), *Prevotella disiens* (medium)
STA44	Wound supperficial	*Streptococcus agalactiae* (65.5%), *Finegoldia* sp. (10.2%), *Anaerococcus* sp. (3.4%), *Actinotignum* sp. (0.5%), *Peptoniphilus* sp. (0.4%), *Actinomyces* sp. (0.1%), *Enterococcus* sp. (0.1%), Unidentified species (19.9%)	***Staphylococcus aureus*** (large), *Streptococcus agalactiae* (large)
STA45	Swab ulcus dig.	***Staphylococcus aureus*** (63.4%), *Streptococcus oralis* (23.4%), ***Staphylococcus*** **sp**. (7.0%), *Finegoldia magna* (3.5%), *Pseudomonas aeruginosa* (0.4%), *Anaerococcus murdochii* (0.2%), *Corynebacterium striatum* (0.2%), *Actinomyces neuii* (0.2%), Unidentified species (1.8%)	***Staphylococcus aureus*** (medium), *Pseudomonas aeruginosa* (medium)

*All detected Staphylococcus species are indicated in bold*.

### The Discrepancy Analysis of Identification Results in Clinical Samples

Twenty four samples with both consistent and discrepant *S. aureus* identification by culture and NGS-based approach were selected for the 16S rRNA and *S. aureus* real-time PCR (Table 8). For all selected samples the 16S rRNA assay revealed presence of bacterial DNA and the *S. aureus* real-time PCR revealed the presence of *S. aureus* DNA between Ct = 18 and Ct = 33.1, and Ct = 21.58 and Ct = 36, respectively. Comparing the results of the differences in Ct values (Δ Ct) between 16S rRNA and *S. aureus* PCR, samples were divided into 3 groups. For the first one (STA2, STA4, STA5, STA6, STA9, STA12, STA32, STA34, STA35, STA36, STA37, STA42, and STA45) the NGS-based approach was able to detect *S. aureus* at high level between 24.3 and 100% (reads in a sample) and the Δ Ct values were the lowest counting from 0.03 to 2.42. The second group included samples (STA1, STA3, STA13, STA33, STA38, and STA39) for which the Δ Ct values varied from 0.85 to 16.58 and the *S. aureus* was detected at the bottom level of 0.2% to maximum 8.5%. The third group consisted of the samples for which the NGS-based approach failed to identify the *S. aureus* (STA7, STA31, STA41, STA43, and STA44). For this group the Δ Ct values were very high at the level between 7.08 and 11.32. Therefore, a lack of *S. aureus* detection with NGS-based approach was a result of a low ratio between *S. aureus* DNA and total bacterial DNA in a sample.

**Table 8:** The discrepancy analysis of 24 clinical samples with real-time PCR.

**Sample**	***S. aureus* PCR (Ct)**	**16S rDNA (Ct)**	**Δ Ct**	***S. aureus* identification with NGS of 16S-23S rRNA region (% of total reads)**	***S. aureus* identification with conventional culture and MALDI-TOF Vitek MS identification (growth)**
STA4	25.32	25.04	0.28	*Staphylococcus aureus* (100%)	*Staphylococcus aureus* (medium)
STA36	24.31	23.86	0.45	*Staphylococcus aureus* (100%)	*Staphylococcus aureus* (≥10e5 CFU/ml.)
STA32	25.23	23.70	1.53	*Staphylococcus aureus* (98.4%)	*Staphylococcus aureus* (medium)
STA35	21.58	20.91	0.67	*Staphylococcus aureus* (98.4%)	*Staphylococcus aureus* (≥10e5 CFU/ml.)
STA6	25.72	25.69	0.03	*Staphylococcus aureus* (95.5%)	*Staphylococcus aureus* (medium)
STA34	28.26	29.70	1.44	*Staphylococcus aureus* (68.7%)	*Staphylococcus aureus* (≥10e5 CFU/ml.)
STA37	27.01	24.59	2.42	*Staphylococcus aureus* (62.4%)	*Staphylococcus aureus* (large)
STA2	27.78	27.20	0.58	*Staphylococcus aureus* (65.3%)	*Staphylococcus aureus* (medium)
STA45	26.26	25.51	0.75	*Staphylococcus aureus* (63.4%)	*Staphylococcus aureus* (medium)
STA5	31.83	32.86	1.03	*Staphylococcus aureus* (59.6%)	*Staphylococcus aureus* (medium)
STA12	25.25	26.72	1.53	*Staphylococcus aureus* (55.0%)	*Staphylococcus aureus* (medium)
STA42	31.29	33.1	1.81	*Staphylococcus aureus* (30.8%)	*Staphylococcus aureus* (medium)
STA9	26.49	25.87	0.62	*Staphylococcus aureus* (24.3%)	*Staphylococcus aureus* (medium)
STA39	25.13	19.22	5.91	*Staphylococcus aureus* (8.5%)	*Staphylococcus aureus* (large)
STA3	31.30	30.45	0.85	*Staphylococcus aureus* (7.8%)	*Staphylococcus aureus* (few)
STA38	26.73	18.00	8.73	*Staphylococcus aureus* (2.7%)	*Staphylococcus aureus* (≥10e5 CFU/ml.)
STA13	34.79	18.21	16.58	*Staphylococcus* sp. (0.9%)	*Staphylococcus haemolyticus* (sporadic), *Staphylococcus epidermidis* (large)
STA33	26.86	25.15	1.71	*Staphylococcus aureus* (0.4%)	*Staphylococcus aureus* (large)
STA1	33.10	21.86	11.15	*Staphylococcus aureus* (0.2%)	*Staphylococcus aureus* (large)
STA7	36.00	28.01	7.99	*Staphylococcus aureus* (0.0%)	*Staphylococcus aureus* (few)
STA31	31.43	21.83	9.60	*Staphylococcus aureus* (0.0%)	*Staphylococcus aureus* (medium)
STA41	33.48	23.12	10.36	*Staphylococcus aureus* (0.0%)	*Staphylococcus aureus* (few)
STA43	28.08	21.00	7.08	*Staphylococcus aureus* (0.0%)	*Staphylococcus aureus* (large)
STA44	31.43	20.11	11.32	*Staphylococcus aureus* (0.0%)	*Staphylococcus aureus* (large)

## Discussion

Because of the increasing clinical significance of CoNS (Becker et al., 2014), accurate identification of staphylococci at the species level is highly desirable to permit a more precise determination of host-pathogen relationships and to better understand the pathogenic potential of various staphylococcal species. Phenotypic identification of CoNS appears to be unsatisfactory, unreliable, and irreproducible (Heikens et al., 2005; Dupont et al., 2010; Bergeron et al., 2011; Singhal et al., 2015; Ayeni et al., 2017). Therefore, applying genetic methods in standard microbiological diagnostics is necessary to improve the identification process. When an unknown organism needs to be identified in a clinical sample, 16S rRNA gene sequencing is the method of choice because of the availability of universal primers (Clarridge, 2004). The 16S rRNA gene sequencing is an appropriate target for most staphylococcal species; however, for some species, inter-species differentiation is difficult or impossible due to missing or insufficient heterogeneity within the 16S rRNA gene. Most reports show that the discriminatory power of 16S rRNA gene sequencing is very low for closely related *Staphylococcus* species (Heikens et al., 2005; Mellmann et al., 2006; Woo et al., 2009; Shin et al., 2011; Lange et al., 2015). Moreover, the accuracy of identification of bacterial species with 16S rRNA gene sequencing is hindered by the low quality of many of the sequences deposited in public databases (Becker et al., 2004). Other targeted sequencing methods may have a higher identification potential than 16S rRNA gene sequencing but often are limited to selected genera (Li et al., 2012). This study showed *sodA* and *rpoB* targets were the most discriminative but NGS of the 16S-23S rRNA region was more discriminative than *tuf* gene sequencing and much more discriminative than 16S rRNA gene sequencing based on obtained sequences and whole database search. Moreover, the NGS-based method showed the same clustering as the other methods (Figure 1). Because NGS of the 16S-23S rRNA region uses universal primers, this method is applicable to different and genetically unrelated bacterial genera.

Beyond the comparison of the five sequence-based methods used for staphylococcal identification, the main purpose of this study was to develop and validate a complete staphylococcal reference sequence dataset for the 16S-23S rRNA region and to evaluate the potential of this method for clinical samples. The NGS of the 16S-23S rRNA region developed by Sabat et al. (2017) provides the ability to detect microorganisms not only in samples from mixed infections, which also consist of commensal microorganisms, but also in whole microbiomes. However, this method suffers from a lack of reference sequences in the GenBank database for many bacterial species at present. Prior to this study, 16S-23S rRNA sequences were available for only 29 *Staphylococcus* species. Our investigations allowed development of 16S-23S rRNA sequences for an additional 21 species, making identification of almost all *Staphylococcus* species feasible with the exception only of the recently described Antarctic *S. edaphicus* species (Pantøuček et al., 2017).

In order to identify strains at the species level, the reference sequence with the highest similarity score needs to be found. For several *Staphylococcus* species, only one or a few reference 16S-23S rRNA sequences can be found during BLAST searches in the GenBank database. In such cases, it is possible that the sequence obtained during a study belongs to a different evolutionary cluster within a species than the reference and the nucleotide differences between them are high (more than 1%). Then, it is not possible to assign bacterial species with the similarity score 99% or higher. If more reference sequences are deposited in the genetic sequence databases, representing evolutionary diverse lineages, species will always be assigned with a similarity score above 99%.

The NGS of 16S-23S rRNA approach proved to be an excellent tool for identification at the species level for a great majority of *Staphylococcus* strains. Nevertheless, some problematic cases were found. In our study, in case of pairs: *S. pseudintermedius – S. aureus; S. simulans – S. hyicus* and *S. warneri – S. epidermidis*, an alignment to the next closest species accounted <0.2% but to only one and not published genome assembly. In all these cases, the second next species was aligned <99% similarity. Similar situation was found for *S. agnetis – S. hyicus* and *S. schweitzeri – S. aureus*. As the problems in accurate identification of these species are described (Tong et al., 2015; Adkins et al., 2017), we believe that the increase of deposited sequences for *S. agnetis* and *S. schweitzeri* will allow for an unequivocal identification. It is very important to develop a well-curated database with a verification of deposited sequences in terms of proper organism identification. For now, the sequences that are not published should not be considered as reference ones. There is no previous single study with a same dataset of reference sequences for genes commonly used for staphylococci identification, so usually those sequences cannot be compared. In this study, we have not only deposited such dataset for 4 commonly used identification targets but also added a package of sequences for a new identification tool with a high identification potential.

The developed reference dataset improved the identification accuracy of staphylococci in clinical samples. Data from this study showed that NGS of the 16S-23S rRNA region for most clinical samples correctly identified the bacterial species that were identified using culture-based methods. In most samples, a few more species of the same genera or other genera were also identified by NGS. In some samples, NGS allowed identification of clinically relevant pathogens that often remain unidentified by culture methods due to their challenging growth, such as GPAC (Gram-positive anaerobic cocci) bacteria, *Actinotignum* sp., and *Streptococcus* species (Murphy and Frick, 2013; Pedersen et al., 2017). In other cases, the NGS results were not consistent with the culture methods. In some cases, the NGS showed the dominating unidentified species within the samples, and in others, a low-quality PCR product was obtained. These problems may be removed by obtaining more reference sequences for other genera and using the NGS method for more clinical samples to slightly improve the PCR conditions. Moreover, when differences can be found within the whole 16S-23S rRNA region sequence, then polymorphism within the species is the cause, and inclusion of a higher number of reference sequences will improve the species-level identification. Facilitating NGS 16S-23S rRNA data analyses is crucial because at present, this method is quite complicated for clinical samples since it is time consuming and the results may be difficult to interpret. The new software that is being created will be a great improvement for researchers. Importantly, positive and negative controls should always be included to monitor the whole process.

The rapid development of DNA sequencing techniques has allowed substantial improvement of culture-independent identification of microbial pathogens. Additionally, advances in DNA sequencing techniques have allowed simultaneous investigation of millions of DNA fragments and enabled rapid identification of all microorganisms present in a given clinical sample. NGS-based techniques, especially NGS of the 16S rRNA gene, have been successfully applied for the comprehensive analysis of microbiomes not only from healthy people but also from microbiomes associated with many diseases (Jervis Bardy and Psaltis, 2016; Jovel et al., 2016; Pérez-Losada et al., 2017). Microbiome analysis could lead to reclassification of terms such as “infectious agent” or “bacterial pathogen,” because any microbiome appears to have one or more dominant bacteria but also contains potentially important coconspirators that may modulate growth, virulence, biofilm formation, quorum sensing, and antibiotic resistance, and sensitive NGS-based techniques enable their detection (Toma et al., 2014). In any case, identification of microbiome constituents at the phylum or genus level does not provide sufficient microbiological details. This lack occurs because microbes at the species level are transmitted between hosts and have different transmission power, tenacity and biological behavior. NGS of the 16-23S rRNA region allows polymicrobial diagnostics, and analysis of the intergenic regions contained in this region significantly increases the identification potential of the method, allowing unambiguous species identification.

In conclusion, our study demonstrated the reliability of NGS of the 16S-23S rRNA region for staphylococcal identification at the species level. The method based on NGS of the 16S-23S region undoubtedly had the highest identification potential of all of the methods used. We have developed a reference dataset of the 16S-23S rRNA region for 50 staphylococcal species (including one proposed species) and 6 subspecies. Therefore, all clinically relevant staphylococcal species can be detected in patient specimens at present. Expanding the database in the future will allow this approach to constitute a highly precise, rapid and reliable method for highly specific microbial identification in general.

## Data Availability

The datasets generated for this study can be found in Genbank, MF678861–MF678916, MK015765-MK015864; MF678917-MF678972, MF679029-MF679084; MF678973-MF679028; MF679085-MF679140.

## Author Contributions

AS, AK-S, and AF designed the project. AK-S, KB, and JM provided the strains and clinical samples with their data. MK-S, VA, EvZ, GW, and AS performed the experiments. MK-S and AS carried out *de novo* assemblies. All authors interpreted the data. MK-S and AS wrote the manuscript. All authors reviewed the manuscript.

### Conflict of Interest Statement

The authors declare that the research was conducted in the absence of any commercial or financial relationships that could be construed as a potential conflict of interest.
