# Supplementary material for: Development and Validation of a Reference Data Set for Assigning Staphylococcus Species Based on Next-Generation Sequencing of the 16S-23S rRNA Region
Source: Front Cell Infect Microbiol. 2019 Aug 7;9:278. doi: 10.3389/fcimb.2019.00278 (PMC6698797; doi:10.3389/fcimb.2019.00278)

**Supplementary Table 1**

Summary of the additional strains identification at the species level.

| **Strain number (Reference collection strain number)** | **Species (routine diagnostic methods)** | **Isolation** | **Routine diagnostic identification method** | **Species after 16S rRNA gene Sanger sequencing** | **Species after NGS 16S-23S** |
| --- | --- | --- | --- | --- | --- |
| **PL456** | *S. arlettae* | animal infection | MALDI-TOF Microflex LT; Bruker | *Staphylococcus* *arlettae* 1283/1283(100%) | *S. arlettae* |
| **PL388 (PCM 2528)** | *S. arlettae* | reference collection | *sodA, rpoB; tuf* | *Staphylococcus* *arlettae* 1287/1287(100%) | *S. arlettae* |
| **PL486** | *S. capitis* | human infection | BD Phoenix™ system | *Staphylococcus* *capitis* 1283/1283(100%)/ *Staphylococcus* *epidermidis* 1283/1283(100%) | *S. capitis* |
| **PL489** | *S. capitis* | healthy animal | API STAPH | *Staphylococcus* *capitis* 1283/1283(100%)/ *Staphylococcus* *epidermidis* 1283/1283(100%) | *S. capitis* |
| **PL492** | *S. capitis* | human infection | BD Phoenix™ system | *Staphylococcus* *capitis* 1283/1283(100%)/ *Staphylococcus* *epidermidis* 1282/1283(99.9%) | *S. capitis* |
| **PL467** | *S. capitis* | human infection | **MALDI-TOF VITEK® MS; BioMeriéux** | *Staphylococcus* *capitis* 1283/1283(100%)/ *Staphylococcus* *epidermidis* 1283/1283(100%) | *S. capitis* |
| **PL488** | *S. capitis* | human infection | VITEK® 2 ID | *Staphylococcus* *epidermidis* 1283/1283(100%)/ *Staphylococcus* *caprae* 1283/1283(100%) | *S. epidermidis* |
| **PL493** | *S. capitis* | human infection | BD Phoenix™ system | *Staphylococcus* *epidermidis* 1282/1283(99.9%)/ *Staphylococcus* *caprae* 1282/1283(99.9%) | *S. epidermidis* |
| **PL490** | *S. capitis* | human infection | **MALDI-TOF VITEK® MS; BioMeriéux** | *Staphylococcus* *warneri* 1282/1283(99.9%)/ *Staphylococcus* *pasteuri* 1282/1283(99.9%) | *S. pasteuri* |
| **PL516** | *S. caprae* | animal infection | MALDI-TOF Microflex LT; Bruker | *Staphylococcus* *caprae* 1283/1283(100%)/ *Staphylococcus* *capitis* 1282/1283(99.9%) | *S. caprae* |
| **PL542** | *S. caprae* | human infection | **MALDI-TOF VITEK® MS; BioMeriéux** | *Staphylococcus* *caprae* 1283/1283(100%)/ *Staphylococcus* *capitis* 1282/1283(99.9%) | *S. caprae* |
| **PL543** | *S. caprae* | human infection | **MALDI-TOF VITEK® MS; BioMeriéux** | *Staphylococcus* *caprae* 1283/1283(100%)/ *Staphylococcus* *capitis* 1282/1283(99.9%) | *S. caprae* |
| **PL544** | *S. caprae* | human infection | **MALDI-TOF VITEK® MS; BioMeriéux** | *Staphylococcus* *caprae* 1283/1283(100%)/ *Staphylococcus* *capitis* 1282/1283(99.9%) | *S. caprae* |
| **PL545** | *S. caprae* | human infection | **MALDI-TOF VITEK® MS; BioMeriéux** | *Staphylococcus* *caprae* 1283/1283(100%)/ *Staphylococcus* *capitis* 1282/1283(99.9%) | *S. caprae* |
| **PL376 (PCM 2455)** | *S. caprae* | reference collection | *sodA, rpoB; tuf* | *Staphylococcus* *caprae* 1282/1283(99.9%)/ *Staphylococcus* *capitis* 1280/1282(99.8%) | *S. caprae* |
| **PL515** | *S. caprae* | human infection | VITEK® 2 ID | *Staphylococcus* *pasteuri* 1283/1283(100%)/ *Staphylococcus* *warneri* 1283/1283(100%) | *S. pasteuri* |
| **PL541** | *S. caprae* | human infection | **MALDI-TOF VITEK® MS; BioMeriéux** | *Staphylococcus* *epidermidis* 1283/1283(100%) | *S. epidermidis* |
| **PL377 (PCM 2429)** | *S. carnosus* | reference collection | *sodA, rpoB; tuf* | *Staphylococcus* *carnosus* 1282/1282(100%)/ *Staphylococcus* *condimenti* 1277/1279(99.8%) | *S. carnosus* |
| **PL442** | *S. chromogenes* | animal infection | MALDI-TOF Microflex LT; Bruker | *Staphylococcus* *chromogenes* 1279/1279(100%) | *S. chromogenes* |
| **PL436** | *S. cohnii* | animal infection | MALDI-TOF Microflex LT; Bruker | *Staphylococcus* *cohnii* 1283/1283(100%) | *S. cohnii* |
| **PL453** | *S. equorum* | natural environment | API STAPH | *Staphylococcus* *haemolyticus* 1283/1283(100%)/ *Staphylococcus* *equorum* 1283/1283(100%) | *S. equorum* |
| **PL454** | *S. equorum* | natural environment | API STAPH | *Staphylococcus* *haemolyticus* 1283/1283(100%)/ *Staphylococcus* *equorum* 1283/1283(100%) | *S. equorum* |
| **PL455** | *S. equorum* | animal infection | MALDI-TOF Microflex LT; Bruker | *Staphylococcus* *haemolyticus* 1283/1283(100%)/ *Staphylococcus* *equorum* 1283/1283(100%) | *S. equorum* |
| **PL452 (LMG 19116)** | *S. equorum* | reference collection | reference collection | *Staphylococcus* *epidermidis* 1283/1283(100%) | *S. epidermidis* |
| **PL393 (PCM 2408)** | *S. felis* | reference collection | *sodA, rpoB; tuf* | *Staphylococcus* *felis* 1282/1283(99.9%) | *S. felis* |
| **PL435** | *S. felis* | animal infection | MALDI-TOF Microflex LT; Bruker | *Staphylococcus* *equorum* 1283/1283(100%)/ *Staphylococcus* *haemolyticus* 1283/1283(100%) | *S. equorum* |
| **PL438** | *S. fleuretti* | infection animal | MALDI-TOF Microflex LT; Bruker | *Staphylococcus* *fleurettii* 1284/1284(100%) | *S. fleuretti* |
| **PL451 (LMG 19119)** | *S. gallinarum* | reference collection | reference collection | *Staphylococcus* *gallinarum* 1283/1283(100%) | *S. gallinarum* |
| **PL394 (PCM 2439)** | *S. gallinarum* | reference collection | *sodA, rpoB; tuf* | *Staphylococcus* *haemolyticus* 1282/1282(100%)/ *Staphylococcus* *gallinarum* 1281/1281(100%) | *S. gallinarum* |
| **PL496** | *S. haemolyticus* | human infection | VITEK® 2 ID | *Staphylococcus* *haemolyticus* 1283/1283(100%)/ *Staphylococcus* *epidermidis* 1282/1283(99.9%) | *S. haemolyticus* |
| **PL499** | *S. haemolyticus* | human infection | VITEK® 2 ID | *Staphylococcus* *haemolyticus* 1283/1283(100%)/ *Staphylococcus* *epidermidis* 1282/1283(99.9%) | *S. haemolyticus* |
| **PL500** | *S. haemolyticus* | healthy animal | API STAPH | *Staphylococcus* *haemolyticus* 1283/1283(100%)/ *Staphylococcus* *epidermidis* 1282/1283(99.9%) | *S. haemolyticus* |
| **PL501** | *S. haemolyticus* | human infection | BD Phoenix™ system | *Staphylococcus* *haemolyticus* 1283/1283(100%)/ *Staphylococcus* *epidermidis* 1282/1283(99.9%) | *S. haemolyticus* |
| **PL503** | *S. haemolyticus* | human infection | BD Phoenix™ system | *Staphylococcus* *haemolyticus* 1283/1283(100%)/ *Staphylococcus* *epidermidis* 1282/1283(99.9%) | *S. haemolyticus* |
| **PL546** | *S. haemolyticus* | human infection | **MALDI-TOF VITEK® MS; BioMeriéux** | *Staphylococcus* *haemolyticus* 1283/1283(100%)/ *Staphylococcus* *epidermidis* 1282/1283(99.9%) | *S. haemolyticus* |
| **PL565** | *S. haemolyticus* | human infection | **MALDI-TOF VITEK® MS; BioMeriéux** | *Staphylococcus* *haemolyticus* 1283/1283(100%)/ *Staphylococcus* *epidermidis* 1282/1283(99.9%) | *S. haemolyticus* |
| **PL547** | *S. haemolyticus* | human infection | **MALDI-TOF VITEK® MS; BioMeriéux** | *Staphylococcus* *epidermidis* 1283/1283(100%) | *S. epidermidis* |
| **PL470** | *S. hominis* | human infection | BD Phoenix™ system | *Staphylococcus* *hominis* 1283/1283(100%) | *S. hominis* |
| **PL472** | *S. hominis* | healthy animal | API STAPH | *Staphylococcus* *hominis* 1283/1283(100%) | *S. hominis* |
| **PL477** | *S. hominis* | human infection | BD Phoenix™ system | *Staphylococcus* *hominis* 1283/1283(100%) | *S. hominis* |
| **PL549** | *S. hominis* | human infection | **MALDI-TOF VITEK® MS; BioMeriéux** | *Staphylococcus* *hominis* 1283/1283(100%) | *S. hominis* |
| **PL471** | *S. hominis* | human infection | VITEK® 2 ID | *Staphylococcus* *epidermidis* 1283/1283(100%)/ *Staphylococcus* *caprae* 1283/1283(100%) | *S. epidermidis* |
| **PL474** | *S. hominis* | human infection | VITEK® 2 ID | *Staphylococcus* *epidermidis* 1283/1283(100%) | *S. epidermidis* |
| **PL548** | *S. hominis* | human infection | **MALDI-TOF VITEK® MS; BioMeriéux** | *Staphylococcus* *haemolyticus* 1283/1283(100%)/ *Staphylococcus* *epidermidis* 1282/1283(99.9%) | *S. haemolyticus* |
| **PL562** | *S. hominis* | human infection | **MALDI-TOF VITEK® MS; BioMeriéux** | *Staphylococcus* *hominis* 1282/1283(99.9%) | *S. hominis* |
| **PL439** | *S. hyicus* | animal infection | MALDI-TOF Microflex LT; Bruker | *Staphylococcus* *agnetis* 1283/1283(100%)/ *Staphylococcus* *hyicus* 1281/1283(99.8%) | *S.agnetis* |
| **PL386 (PCM 2405)** | *S. intermedius* | reference collection | *sodA, rpoB; tuf* | *Staphylococcus* *intermedius* 1301/1304(99.8%)/ *Staphylococcus* *pseudintermedius* 1300/1304(99.7%) | *S. intermedius* |
| **PL525** | *S. intermedius* | animal infection | API STAPH | *Staphylococcus* *pseudintermedius* 1283/1283(100%)/ *Staphylococcus* *intermedius* 1282/1283(99.9%) | *S. pseudintermedius* |
| **PL526** | *S. intermedius* | animal infection | API STAPH | *Staphylococcus* *pseudintermedius* 1283/1283(100%)/ *Staphylococcus* *intermedius* 1282/1283(99.9%) | *S. pseudintermedius* |
| **PL527** | *S. intermedius* | animal infection | API STAPH | *Staphylococcus* *pseudintermedius* 1283/1283(100%)/ *Staphylococcus* *intermedius* 1282/1283(99.9%) | *S. pseudintermedius* |
| **PL529** | *S. intermedius* | animal infection | API STAPH | *Staphylococcus* *pseudintermedius* 1283/1283(100%)/ *Staphylococcus* *intermedius* 1282/1283(99.9%) | *S. pseudintermedius* |
| **PL530** | *S. intermedius* | animal infection | API STAPH | *Staphylococcus* *pseudintermedius* 1283/1283(100%)/ *Staphylococcus* *intermedius* 1282/1283(99.9%) | *S. pseudintermedius* |
| **PL457** | *S. kloosii* | human infection | BD Phoenix™ system | *Staphylococcus* *aureus* 1282/1283(99.9%)/ *Staphylococcus* *argenteus* 1282/1283(99.9%) | *S. aureus* |
| **PL458** | *S. kloosii* | animal infection | MALDI-TOF Microflex LT; Bruker | *Staphylococcus* *equorum* 1283/1283(100%)/ *Staphylococcus* *haemolyticus* 1283/1283(100%) | *S. equorum* |
| **PL444** | *S. lentus* | natural environment | API STAPH | *Staphylococcus* *lentus* 1284/1284(100%) | *S. lentus* |
| **PL445** | *S. lentus* | natural environment | API STAPH | *Staphylococcus* *lentus* 1284/1284(100%) | *S. lentus* |
| **PL447** | *S. lugdunensis* | human infection | VITEK® 2 ID | *Staphylococcus* *lugdunensis* 1283/1283(100%) | *S. lugdunensis* |
| **PL448** | *S. lugdunensis* | human infection | VITEK® 2 ID | *Staphylococcus* *lugdunensis* 1283/1283(100%) | *S. lugdunensis* |
| **PL551** | *S. lugdunensis* | human infection | **MALDI-TOF VITEK® MS; BioMeriéux** | *Staphylococcus* *lugdunensis* 1283/1283(100%) | *S. lugdunensis* |
| **PL552** | *S. lugdunensis* | human infection | **MALDI-TOF VITEK® MS; BioMeriéux** | *Staphylococcus* *lugdunensis* 1283/1283(100%) | *S. lugdunensis* |
| **PL553** | *S. lugdunensis* | human infection | **MALDI-TOF VITEK® MS; BioMeriéux** | *Staphylococcus* *lugdunensis* 1283/1283(100%) | *S. lugdunensis* |
| **PL554** | *S. lugdunensis* | human infection | **MALDI-TOF VITEK® MS; BioMeriéux** | *Staphylococcus* *lugdunensis* 1283/1283(100%) | *S. lugdunensis* |
| **PL555** | *S. lugdunensis* | human infection | **MALDI-TOF VITEK® MS; BioMeriéux** | *Staphylococcus* *lugdunensis* 1283/1283(100%) | *S. lugdunensis* |
| **PL550** | *S. lugdunensis* | human infection | **MALDI-TOF VITEK® MS; BioMeriéux** | *Staphylococcus* *lugdunensis* 1282/1283(99.9%) | *S. lugdunensis* |
| **PL446** | *S. lugdunensis* | human infection | VITEK® 2 ID | *Staphylococcus* *hominis* 1283/1283(100%) | *S. hominis* |
| **PL440** | *S. nepalensis* | animal infection | MALDI-TOF Microflex LT; Bruker | *Staphylococcus* *nepalensis* 1283/1283(100%)/ *Staphylococcus* *cohnii* 1283/1283(100%) | *S. nepalensis* |
| **PL556** | *S. pasteuri* | human infection | **MALDI-TOF VITEK® MS; BioMeriéux** | *Staphylococcus* *pasteuri* 1283/1283(100%)/ *Staphylococcus* *warneri* 1283/1283(100%) | *S. pasteuri* |
| **PL557** | *S. pasteuri* | human infection | **MALDI-TOF VITEK® MS; BioMeriéux** | *Staphylococcus* *pasteuri* 1283/1283(100%)/ *Staphylococcus* *warneri* 1283/1283(100%) | *S. pasteuri* |
| **PL449** | *S. pettenkoferi* | human infection | *gap; saoC* | *Staphylococcus* *pettenkoferi* 1283/1283(100%) | *S. pettenkoferi* |
| **PL450** | *S. pettenkoferi* | human infection | BD Phoenix™ system | *Staphylococcus* *capitis* 1282/1283(99.9%)/ *Staphylococcus* *epidermidis* 1282/1283(99.9%) | *S. capitis* |
| **PL531** | *S. pseudintermedius* | animal infection | *gap; rpoB* | *Staphylococcus* *pseudintermedius* 1283/1283(100%)/ *Staphylococcus* *intermedius* 1282/1283(99.9%) | *S. pseudintermedius* |
| **PL534** | *S. pseudintermedius* | animal infection | *gap; rpoB* | *Staphylococcus* *pseudintermedius* 1283/1283(100%)/ *Staphylococcus* *intermedius* 1282/1283(99.9%) | *S. pseudintermedius* |
| **PL535** | *S. pseudintermedius* | animal infection | *gap; rpoB* | *Staphylococcus* *pseudintermedius* 1283/1283(100%)/ *Staphylococcus* *intermedius* 1282/1283(99.9%) | *S. pseudintermedius* |
| **PL536** | *S. pseudintermedius* | human infection | *gap; rpoB* | *Staphylococcus* *pseudintermedius* 1283/1283(100%)/ *Staphylococcus* *intermedius* 1282/1283(99.9%) | *S. pseudintermedius* |
| **PL538** | *S. pseudintermedius* | animal infection | API STAPH | *Staphylococcus* *pseudintermedius* 1283/1283(100%)/ *Staphylococcus* *intermedius* 1282/1283(99.9%) | *S. pseudintermedius* |
| **PL479** | *S. saprophyticus* | human infection | API STAPH | *Staphylococcus* *saprophyticus* 1283/1283(100%)/ *Staphylococcus* *xylosus* 1282/1283(99.9%) | *S. saprophyticus* |
| **PL483** | *S. saprophyticus* | human infection | BD Phoenix™ system | *Staphylococcus* *saprophyticus* 1283/1283(100%)/ *Staphylococcus* *xylosus* 1283/1283(100%) | *S. saprophyticus* |
| **PL484** | *S. saprophyticus* | animal infection | MALDI-TOF Microflex LT; Bruker | *Staphylococcus* *saprophyticus* 1283/1283(100%)/ *Staphylococcus* *xylosus* 1283/1283(100%) | *S. saprophyticus* |
| **PL481** | *S. saprophyticus* | human infection | API STAPH | *Staphylococcus* *aureus* 1282/1283(99.9%)/ *Staphylococcus* *argenteus* 1282/1283(99.9%) | *S. aureus* |
| **PL482** | *S. saprophyticus* | human infection | API STAPH | *Staphylococcus* *haemolyticus* 1283/1283(100%)/ *Staphylococcus* *epidermidis* 1282/1283(99.9%) | *S. haemolyticus* |
| **PL459** | *S. sciuri* | animal infection | API STAPH | *Staphylococcus* *sciuri* 1284/1284(100%)/ *Staphylococcus* *lentus* 1283/1284(99.9%) | *S. sciuri* |
| **PL460** | *S. sciuri* | healthy animal | API STAPH | *Staphylococcus* *sciuri* 1284/1284(100%)/ *Staphylococcus* *lentus* 1284/1284(100%) | *S. sciuri* |
| **PL462** | *S. sciuri* | healthy animal | API STAPH | *Staphylococcus* *sciuri* 1284/1284(100%)/ *Staphylococcus* *lentus* 1284/1284(100%) | *S. sciuri* |
| **PL465** | *S. sciuri* | natural environment | API STAPH | *Staphylococcus* *sciuri* 1284/1284(100%)/ *Staphylococcus* *lentus* 1284/1284(100%) | *S. sciuri* |
| **PL468** | *S. sciuri* | animal infection | MALDI-TOF Microflex LT; Bruker | *Staphylococcus* *sciuri* 1284/1284(100%)/ *Staphylococcus* *lentus* 1283/1284(99.9%) | *S. sciuri* |
| **PL441** | *S. simulans* | animal infection | MALDI-TOF Microflex LT; Bruker | *Staphylococcus* *simulans* 1283/1283(100%) | *S. simulans* |
| **PL558** | *S. simulans* | human infection | **MALDI-TOF VITEK® MS; BioMeriéux** | *Staphylococcus* *simulans* 1282/1283(99.9%) | *S. simulans* |
| **PL559** | *S. simulans* | human infection | **MALDI-TOF VITEK® MS; BioMeriéux** | *Staphylococcus* *simulans* 1282/1283(99.9%) | *S. simulans* |
| **PL443** | *S. succinus* | animal infection | MALDI-TOF Microflex LT; Bruker | *Staphylococcus* *succinus* 1283/1283(100%) | *S. succinus* |
| **PL437** | *S. vitulinus* | animal infection | MALDI-TOF Microflex LT; Bruker | *Staphylococcus* *aureus* 1283/1284(99.9%)/ *Staphylococcus* *vitulinus* 1283/1284(99.9%) | *S. vitulinus* |
| **PL508** | *S. warneri* | human infection | BD Phoenix™ system | *Staphylococcus* *warneri* 1283/1283(100%)/ *Staphylococcus* *pasteuri* 1283/1283(100%) | *S. warneri* |
| **PL513** | *S. warneri* | human infection | BD Phoenix™ system | *Staphylococcus* *warneri* 1283/1283(100%)/ *Staphylococcus* *pasteuri* 1283/1283(100%) | *S. warneri* |
| **PL560** | *S. warneri* | human infection | **MALDI-TOF VITEK® MS; BioMeriéux** | *Staphylococcus* *warneri* 1283/1283(100%)/ *Staphylococcus* *pasteuri* 1282/1283(99.9%) | *S. warneri* |
| **PL561** | *S. warneri* | human infection | **MALDI-TOF VITEK® MS; BioMeriéux** | *Staphylococcus* *warneri* 1282/1283(99.9%)/ *Staphylococcus* *pasteuri* 1282/1283(99.9%) | *S. warneri* |
| **PL383 (PCM 2107)** | *S. warneri* | reference collection | *sodA, rpoB; tuf* | *Staphylococcus* *warneri* 1282/1282(100%)/ *Staphylococcus* *pasteuri* 1282/1282(100%) | *S. warneri* |
| **PL511** | *S. warneri* | human infection | BD Phoenix™ system | *Staphylococcus* *warneri* 1283/1283(100%)/ *Staphylococcus* *pasteuri* 1283/1283(100%) | *S. warneri* |
| **PL506** | *S. warneri* | human infection | VITEK® 2 ID | *Escherichia* *coli* 1276/1285(99.3%)/ *Shigella* *dysenteriae* 1276/1285(99.3%) | *E. coli* |
| **PL509** | *S. warneri* | human infection | BD Phoenix™ system | *Staphylococcus* *aureus* 1283/1283(100%)/ *Staphylococcus* *argenteus* 1283/1283(100%) | *S. aureus* |
| **PL517** | *S. xylosus* | animal infection | API STAPH | *Staphylococcus* *xylosus* 1283/1283(100%)/ *Staphylococcus* *saprophyticus* 1283/1283(100%) | *S. xylosus* |
| **PL519** | *S. xylosus* | natural environment | API STAPH | *Staphylococcus* *xylosus* 1283/1283(100%)/ *Staphylococcus* *saprophyticus* 1283/1283(100%) | *S. xylosus* |
| **PL523** | *S. xylosus* | animal infection | MALDI-TOF Microflex LT; Bruker | *Staphylococcus* *xylosus* 1283/1283(100%)/ *Staphylococcus* *saprophyticus* 1283/1283(100%) | *S. xylosus* |

**Supplementary Figure 1 A-D**

Evolutionary relationships of staphylococci species based on *sodA*, *tuf*, *rpoB* and 16S rRNA gene. The evolutionary history was inferred using the Neighbor-Joining method. The percentage of replicate trees in which the associated taxa clustered together in the bootstrap test (1000 replicates) are shown next to the branches. The tree is drawn to scale, with branch lengths in the same units as those of the evolutionary distances used to infer the phylogenetic tree. The evolutionary distances were computed using the Jukes-Cantor method and are in the units of the number of base substitutions per site. Evolutionary analyses were conducted in MEGA7. The strains which are placed in boxes have grouped together in all methods used.

(A)


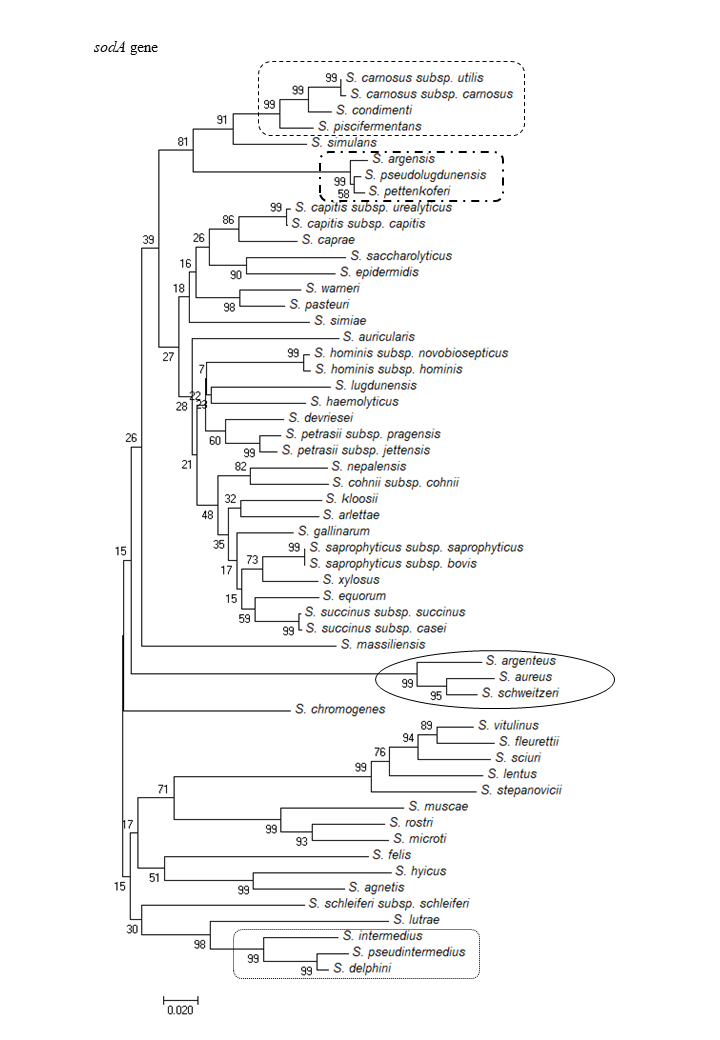


(B)


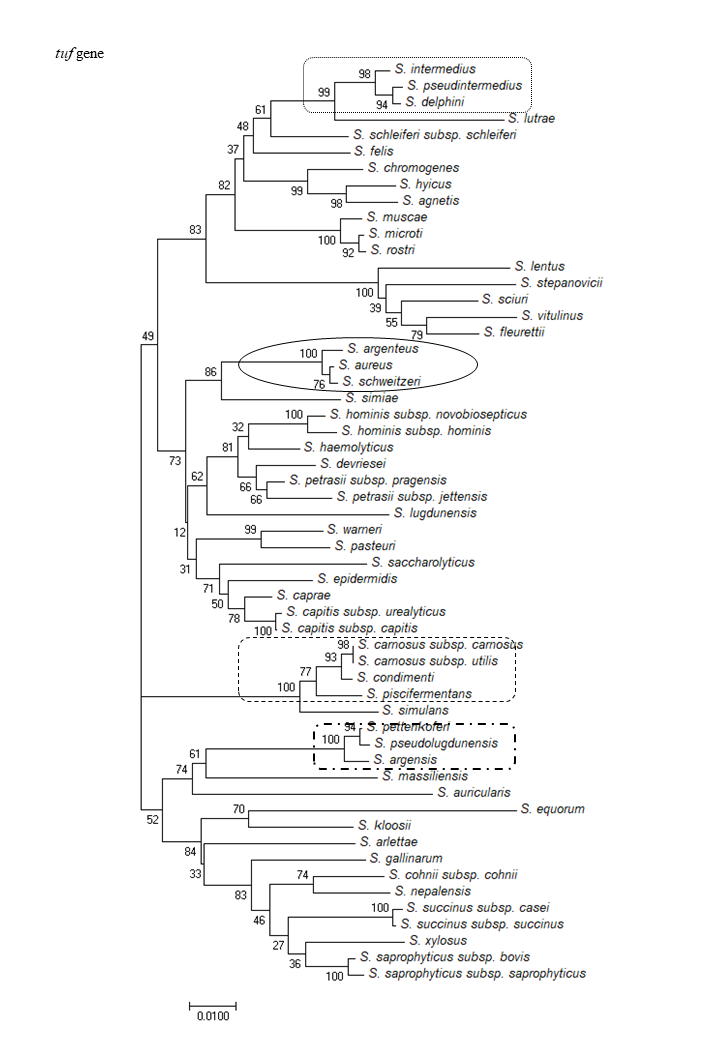


(C)


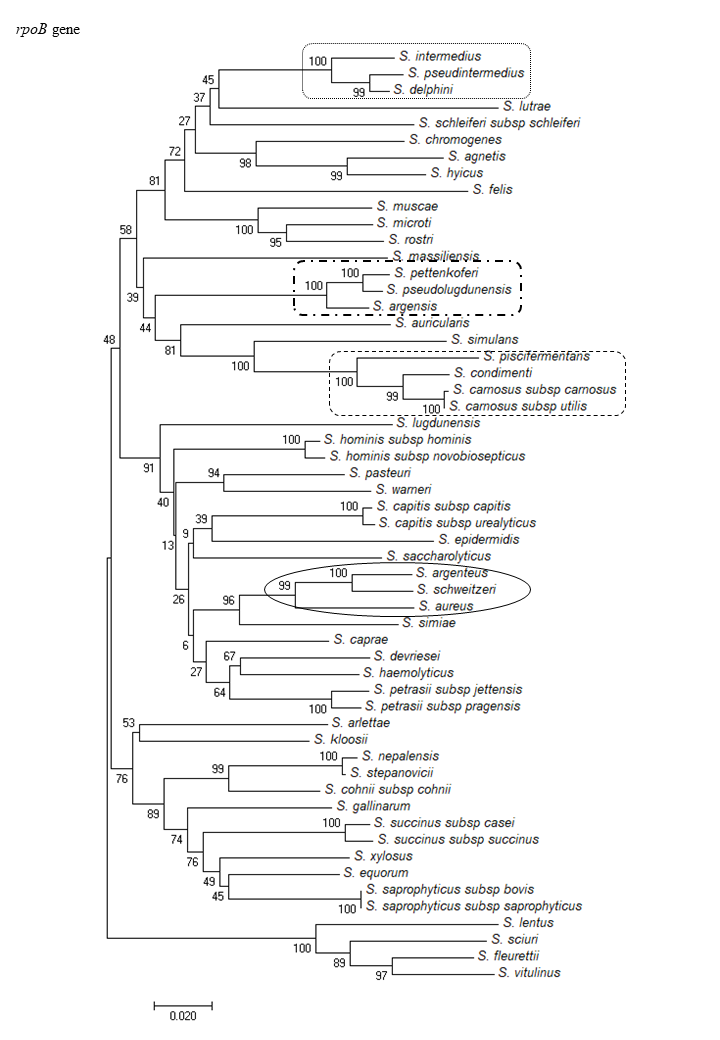


(D)


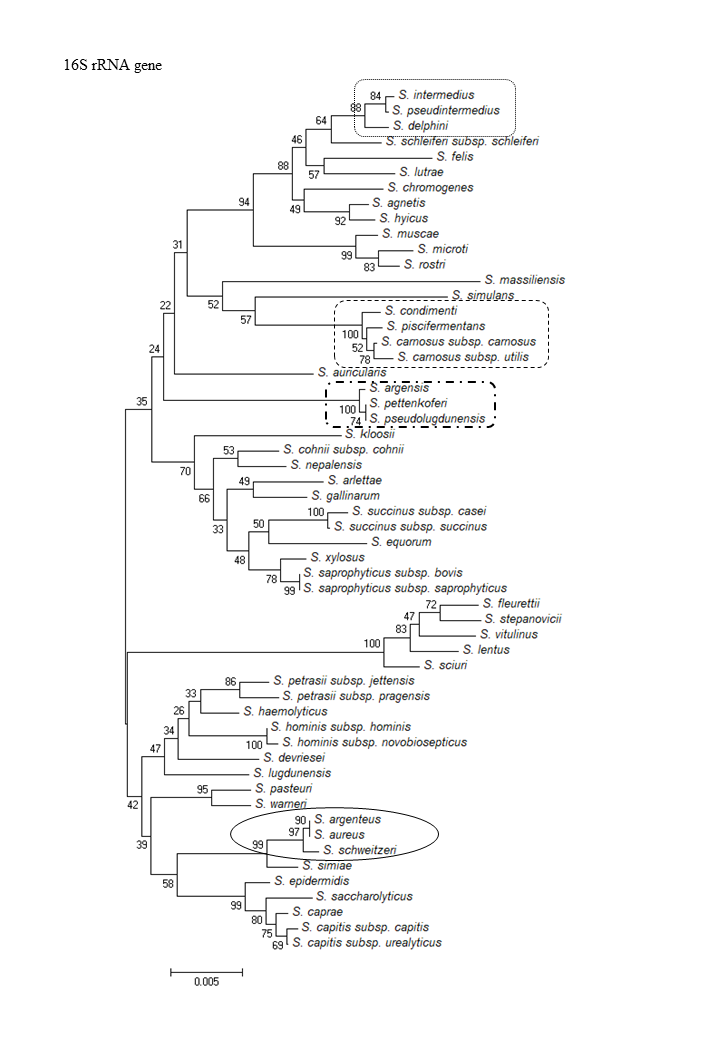

Supplement: Supplementary file 1 [file Data_Sheet_1.docx]
